# In.To. COVID-19 socio-epidemiological co-causality

**DOI:** 10.1038/s41598-022-09656-1

**Published:** 2022-04-06

**Authors:** Elroy Galbraith, Jie Li, Victor J. Del Rio-Vilas, Matteo Convertino

**Affiliations:** 1 Nexus Group, Faculty and Graduate School of Information Science and Technology, Hokkaido University, Sapporo, Japan; 2 Informatics Institute, University of Amsterdam, Amsterdam, The Netherlands; 3 SEARO, World Health Organization, New Delhi, India; 4 fuTuRE EcoSystems Lab, Institute of Environment and Ecology, Tsinghua SIGS, Tsinghua University, Shenzhen, China; 5 Tsinghua Shenzhen International Graduate School, University Town of Shenzhen, Tsinghua Park, Nanshan District Shenzhen, 518055 China

**Keywords:** Infectious diseases, Computational science

## Abstract

Social media can forecast disease dynamics, but infoveillance remains focused on infection spread, with little consideration of media content reliability and its relationship to behavior-driven epidemiological outcomes. Sentiment-encoded social media indicators have been poorly developed for expressed text to forecast healthcare pressure and infer population risk-perception patterns. Here we introduce Infodemic Tomography (InTo) as the first web-based interactive infoveillance cybertechnology that forecasts and visualizes spatio-temporal sentiments and healthcare pressure as a function of social media positivity (i.e., Twitter here), considering both epidemic information and potential misinformation. Information spread is measured on volume and retweets, and the Value of Misinformation (VoMi) is introduced as the impact on forecast accuracy where misinformation has the highest dissimilarity in information dynamics. We validated InTo for COVID-19 in New Delhi and Mumbai by inferring distinct socio-epidemiological risk-perception patterns. We forecast weekly hospitalization and cases using ARIMA models and interpolate spatial hospitalization using geostatistical kriging on inferred risk perception curves between tweet positivity and epidemiological outcomes. Geospatial tweet positivity tracks accurately $\sim$60$\%$ of hospitalizations and forecasts hospitalization risk hotspots along risk aversion gradients. VoMi is higher for risk-prone areas and time periods, where misinformation has the highest non-linear predictability, with high incidence and positivity manifesting popularity-seeking social dynamics. Hospitalization gradients, VoMi, effective healthcare pressure and spatial model-data gaps can be used to predict hospitalization fluxes, misinformation, healthcare capacity gaps and surveillance uncertainty. Thus, InTo is a participatory instrument to better prepare and respond to public health crises by extracting and combining salient epidemiological and social surveillance at any desired space-time scale.

## Introduction

### COVID-19 and infoveillance

The spread and magnitude of COVID-19 is reflected in social media production and sentiments with the lowest ever recorded trend in population positivity (see the Hedonometer at https://hedonometer.org/timeseries/en_all/). Not only are social media messages the saddest they have been since happiness monitoring began (see Dodds et al.^1^), but the volume of misinformation has grown exponentially^2,3^. These observations provide evidence of the relevance of socio-technological systems like social media to predict epidemiology. Empirical evidence for many diseases before COVID-19 and previous analytical findings made clear the linkage between risk perception and infection patterns^4^; thus, highlighting the co-causality of social and epidemiological information beyond their predictability.

Aware of these linkages, global response to COVID-19 by health authorities includes risk communication messages, e.g. on increasing social distancing and using masks to reduce inter-person transmission. Similarly, messages on enhancing early identification, isolation and care for patients all in a bid to “flatten the curve” shed light on the importance of surveillance and public health capacities^5^. The search for social surveillance tools that could help public health officials to monitor, forecast, plan, evaluate and prepare for public health demand started well before COVID, e.g. with seasonal influenza in USA coupled to predictive multimodeling (see Paul et al.^6^, Santillana et al.^7^ and McGowan et al.^8^), due to the recognition of the limitations—e.g. delays, misreporting—of traditional epidemiological surveillance systems. In analogy, social media signals are also used to forecast, a priori or in near real-time, extreme environmental phenomena such as earthquakes^9^, which highlights the relevance of temporal and spatial social media for surveillance.

Concurrently to the spread of COVID-19 epidemic, health authorities are combating an infodemic, strictly defined as the rapid exponential increase in the volume of potentially misleading information about an event^10^. Misinformation, considered as objectively false or inaccurate information, is of difficult detection and classification because it is highly affected by perception bias. Misinformation can tangibly and negatively impact response strategies and health-seeking behaviors^11,12,13^ which may lead to increased infections and hospitalization. Against this background, infodemiology and infoveillance^14^ are strong public health responses to the COVID-19 pandemic and its simultaneous infodemic. *Infodemiology* is the study of the emergent volume, spread, and quality (among other features) of socially-produced information—both accurate and inaccurate—usually related to public health. *Infoveillance* is the surveillance of such social information with health saliency, with the additional aim of detecting and forecasting disease outbreaks that is also the core aim of traditional epidemiological surveillance by using epidemiological data^14^. Both disciplines take behaviors and messages (in text, image, video and sound data) on electronic media, such as the internet as their focus of analysis, along with additional data such as metadata and message sentiments extracted from the primary information. Prior to COVID-19, scientists have been able to use internet dynamics and message sentiments measured as categorical emotions to monitor public health related phenomena and forecast disease spread^15,16,14,17,18,7^. Our work improves on the previous by incorporating a model and information system that uses social media sentiments to forecast sentiments as continuous variables and healthcare pressure (cases and hospitalization) together, over space and time; we combine epidemiology and information patterns, and quantify the effective impact of information, and misinformation alike, on populations.

### Information-prediction nexus

A different perspective on public health forecasting is brought by proposing an assumption-free minimalist model that is focused on patterns rather than processes of the phenomena considered. The employed information-theoretic models (perfectly fitting the general aims of infoveillance) are using the necessary and sufficient social data as sentinels of change, coupled to epidemiological information, to maximize prediction accuracy for the patterns investigated. Information theoretic models like the one proposed here are the least biased models (mechanisms-free) for capturing which set of information is relevant for predicting patterns. Other underlying causal factors, such as local language and socio-environmental factors of the population considered, are certainly important in the domain of physical reality but not in the information domain of predictions. Therefore, the focus is on predictive causality rather than true causality^19^; a principle that, however, should be associated to any model considering the fundamental reality of any model as a microscope of reality rather than its utopian replica.

With the aforementioned reasoning in mind, social and epidemiological processes (and yet data about them) are linked by information and misinformation that is revealing patterns of people behavior in terms of sentiments (informative of risk perception) and cases, respectively. Additionally, strong predictive causality in process-related variables has been shown to coincide with physical causality; yet, computation that screens and weights information can be used to infer co-causality between two signals robustly, without imposing any assumption a priori on model structure.

In the current COVID-19 context, we are interested in knowing whether modern social media are predictive of explosive epidemics, and more precisely which social chatter features are the most predictive of epidemiological patterns. Moreover, whether social chatter features can be accurately used as early warning predictors of risk before cases occur, and how early can forecasts be made. Motivated by these questions we developed InTo as an exploratory tool to quantify how much perceived risk inferred from social chatter in advance was predictive of actual observed risk in cases and extreme cases (or hospitalization) reported by official public health surveillance. This modus operandi and modern infoveillance tool, beyond assessing how much waves in socio- and health-scapes copredict each other via joint “infoscapes”, can validate classical surveillance systems (which provide data that are byproducts of behavioral models, oftentimes affected by strong bias) considering the temporal gap between model and data for multiple surveillance criteria^20^. Theoretically, the smaller the gap over time the higher the surveillance accuracy.

### InTo: infodemic tomography

Infodemic Tomography (In.To. or InTo hereafter) was developed as a cybertechnology to forecast one week in advance COVID-19 related cases, hospitalizations, population positivity, misinformation impact and spreading, healthcare satisfaction and space-time surveillance uncertainty by leveraging geospatial Tweets and epidemiological data in New Delhi and Mumbai as case studies. InTo analyzes and visualizes “tomograms”, as snapshots of epidemiological and information dynamics, for the selected geographies. Thus, InTo is proposed as a pattern-oriented Digital Health platform for Participatory, Predictive, Personalized, Preventive and Precise Health (“P5”), that is an “upgrade” with respect to the “P4” purview of health, such as in Alonso et al.^21^, via the *precise* identification and provision of systemic health-related information to individuals and populations alike. Weather forecasting is the general epitome of InTo considering its focus on predicting patterns of healthcare pressure as a function of dynamically updated information; thus the InTo dashboard is ideally like an App visualizing the most updated weather forecasts.

Previous efforts have focused on internet-based social media for incidence surveillance and outbreak forecasting^22^. Some of these efforts incorporated hospital visit data in their models^23^ but none of them coupled social and epidemiological or healthcare information together. Other process-based models, e.g. Kastalskiy et al.^24^, have numerically explored the linkage between social stress and COVID-19 infections, but these models explored hypothetical mechanisms through assumed analytics that is not inferred a priori from assumption-free models. Therefore, prediction accuracy of these models is not a ”gold standard” to claim their representativeness of real processes. InTo goes beyond temporal incidence predictions because it aims to investigate changes in socio-epi patterns over time and space, and the value of spatial ”social chatter” by dynamically calibrating the model as data from social and epidemiological surveillance is updated. Note that InTo does not make any assumption but the choice of the model (e.g. ARIMA) is based on inferred socio-epidemiological relationships. In this optic and in relation to the early forecasting nature of InTo, the predicted hospitalization is informative of people potentially in need of hospitalization one week in advance. Gradients of hospitalization over space are indicative of patient hospital loads. In an hydroclimatological analogy, gradients of healthcare pressure are like gradients in atmospheric pressure dictating where ill people/rain will likely flow, and exceedance of pressure over healthcare capacity are like floods.

Considering previous efforts, InTo is the first cyberinfrastructure to forecast COVID-19 specific healthcare pressure (as difference between point- and city-scale predicted cases and hospitalization) as a function of text positivity where the latter is a variable quantifying potential happiness in words shared via social media, i.e. Twitter in this context. Although InTo is not the first to examine the relationship between Twitter sentiments and diseases, previous efforts were based on extracting few categorical emotions or using volume of social media entries as predictive functions^16,25,26,27^. InTo instead is the first effort, set of models and participatory dashboard to use quantitative measures of continuous sentiments (associated also to potential misinformation) as positivity to forecast healthcare pressure over space and one week in advance, coupled to the evaluation of those forecasts within an information-theoretic framework.

In the development of InTo we chose to call happiness, introduced by Dodds et al.^1^. as positivity because it is semantically a more general word that does not imply happiness (strict sensu) and relates more effectively to risk behavioral patterns (related to the objective relative risk conditional to the geographical area considered), at least conceptually. Gradients in positivity as a function of cases or hospitalizations define risk perception patterns on which predictive models are calibrated to produce forecasts. Linear predictive models (selected upon linear socio-epidemiological relationships inferred on weekly data) are used to perform infection case and hospitalization forecasts whose predictive power is tested via non-linear predictability indicators (i.e., Transfer Entropy, TE, measuring the time-delayed uncertainty reduction between positivity and epidemiological information (see Li and Convertino^28^ for details of TE as information flow), as discussed in the Material, Methods and Implementation section). These indicators are based on probability distribution functions of the variables of interest and yet they implicitly consider uncertainty distributions that are also attributable to other unexplained uncertainty sources. In this broad framework, properly calibrated positivity fluctuations are good sentinels of relative hospitalization risks—and yet good predictors—as much as heat index fluctuations are good sentinels of extreme temperature hospitalization^29^, to mention an analogous public health effort focused on detecting optimal indicators for risk communication.

**Figure 1:**
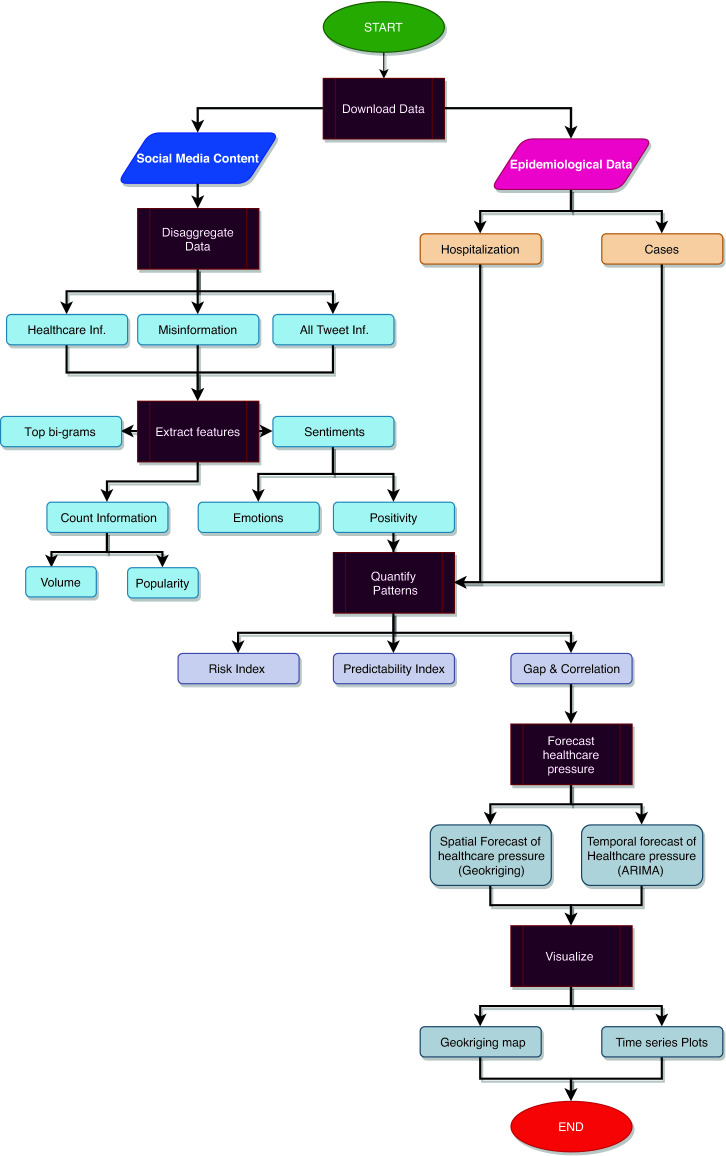
Conceptual and computational workflow of InTo. The process begins with downloading both social media content and epidemiological data. Social media data is then disaggregated into content related to healthcare and misinformation, with the aggregated content retained for analysis as well. As for epidemiological data, the dashboard makes use of hospitalization and cases data for the disease considered. The next process is the extraction of features from social media content: for each subset, bi-grams, count information and sentiments are quantified. Metrics quantifying the relationships between sentiment and epidemiological data are then calculated. Once the linear regression coefficients are estimated (considering the linearity found at the weekly scale between positivity and hospitalization as well as cases; however further non-linear models can be used), these are used to forecast the spatial and temporal variation of healthcare pressure, which is then visualized for users on the dashboard. To illustrate the process and output of InTo we examine the case of New Delhi and Mumbai in India.

The paper presents the workflow in Fig. 1 and implementation of InTo by using the case study of New Delhi and Mumbai to demonstrate its applicability and utility for COVID-19 and in general for any disease. Part of the demonstration includes results of validation exercises conducted to evaluate the developed models. We then discuss limitations of InTo, especially in terms of data availability, representativeness and model complexity. We conclude by outlining future work for InTo.

## Case study results

**Figure 2:**
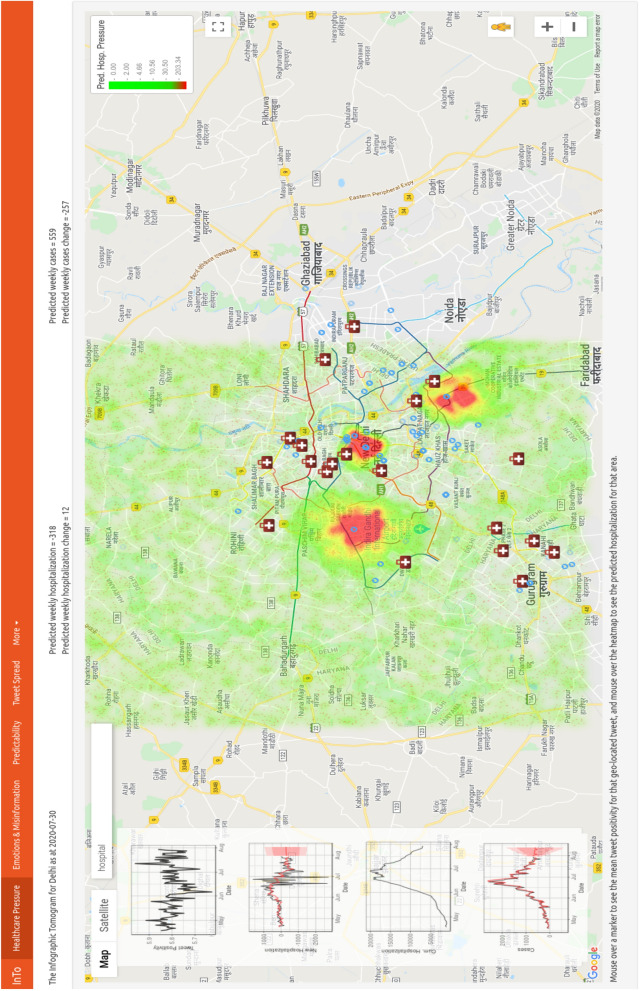
Dashboard Tab 1: healthcare pressure spatial Predictions. Users are presented with a heatmap overlaying satellite imagery obtained from Google Maps, time series of tweet positivity, cases, new hospitalizations and cumulative hospitalizations. In addition, two text outputs inform the user about the predicted hospitalization and cases for the selected city and the difference between the current predicted values and the values predicted for last week. The heatmap visualizes the results of geokriging the spatial interpolation of hospitalization as a function of tweet positivity. Hotter areas are where patients potentially in need of hospitalization are concentrated.

**Figure 3:**
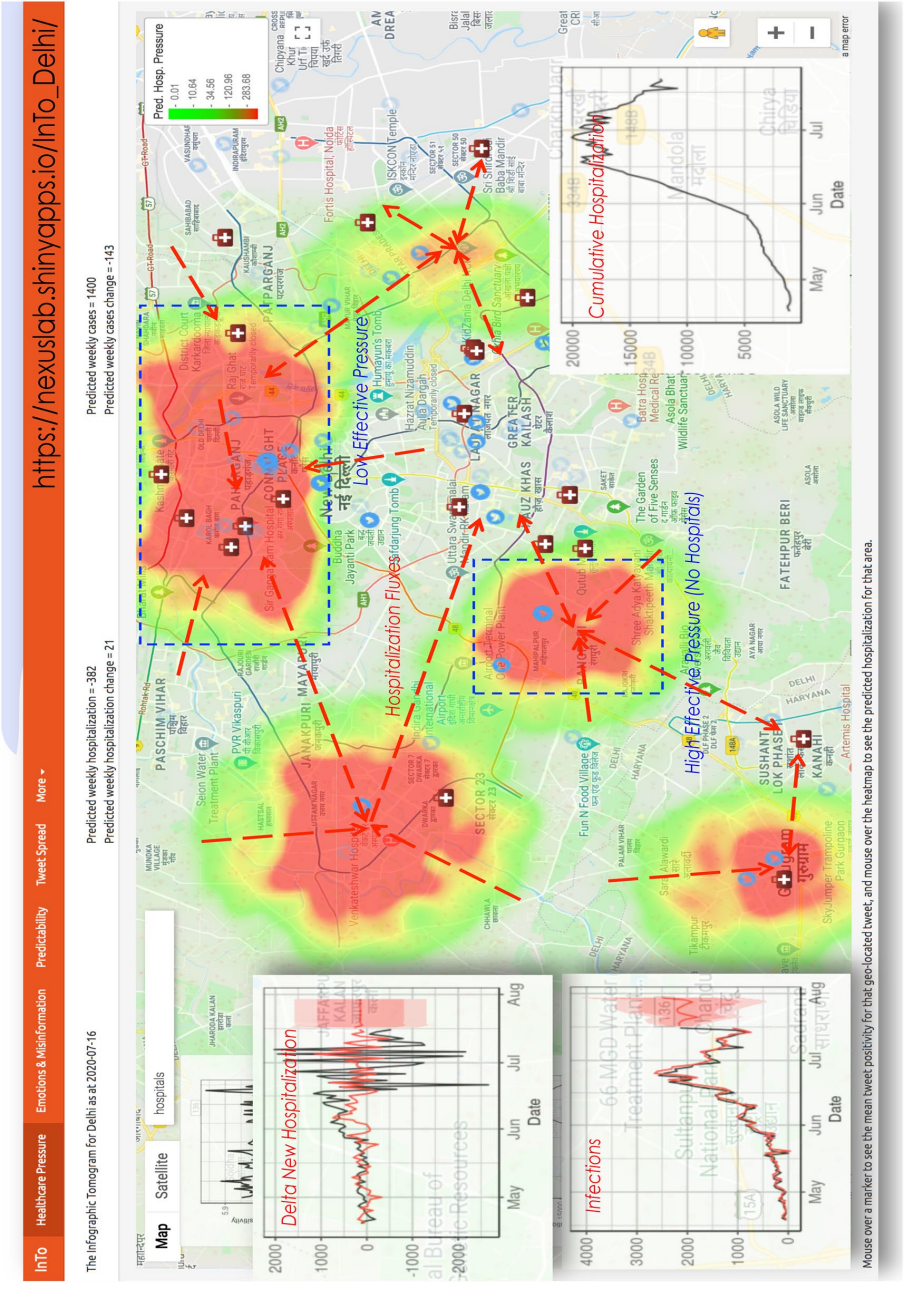
Spatial forecasts. Gradients of Healthcare Pressure, that is $HP_i = \hat{H_{T_i}} - H_T$, reflect potential movement of people in need of hospitalization (hospitalization fluxes). The sum of $HP_i$ from geokriging over space is theoretically equal to the predicted cumulative hospitalization over time ($H_T = \sum _t \Delta H(t)$) as a function of the new hospitalization (that are temporal hospitalization fluxes or healthcare pressure over time). An Effective Healthcare Pressure can be calculated as difference between normalized $HP_i$ and healthcare capacity ($HC_I$) as a function of area healthcare infrastructure resources (e.g. beds, ICUs, ventilators). Uncertainty in forecasts can also consider spatially explicit testing rate and surveillance capacity. In analogy to weather forecasts, gradient of pressure over space are the byproduct of gradient of pressure over time modulated by underlying environmental conditions.

**Figure 4:**
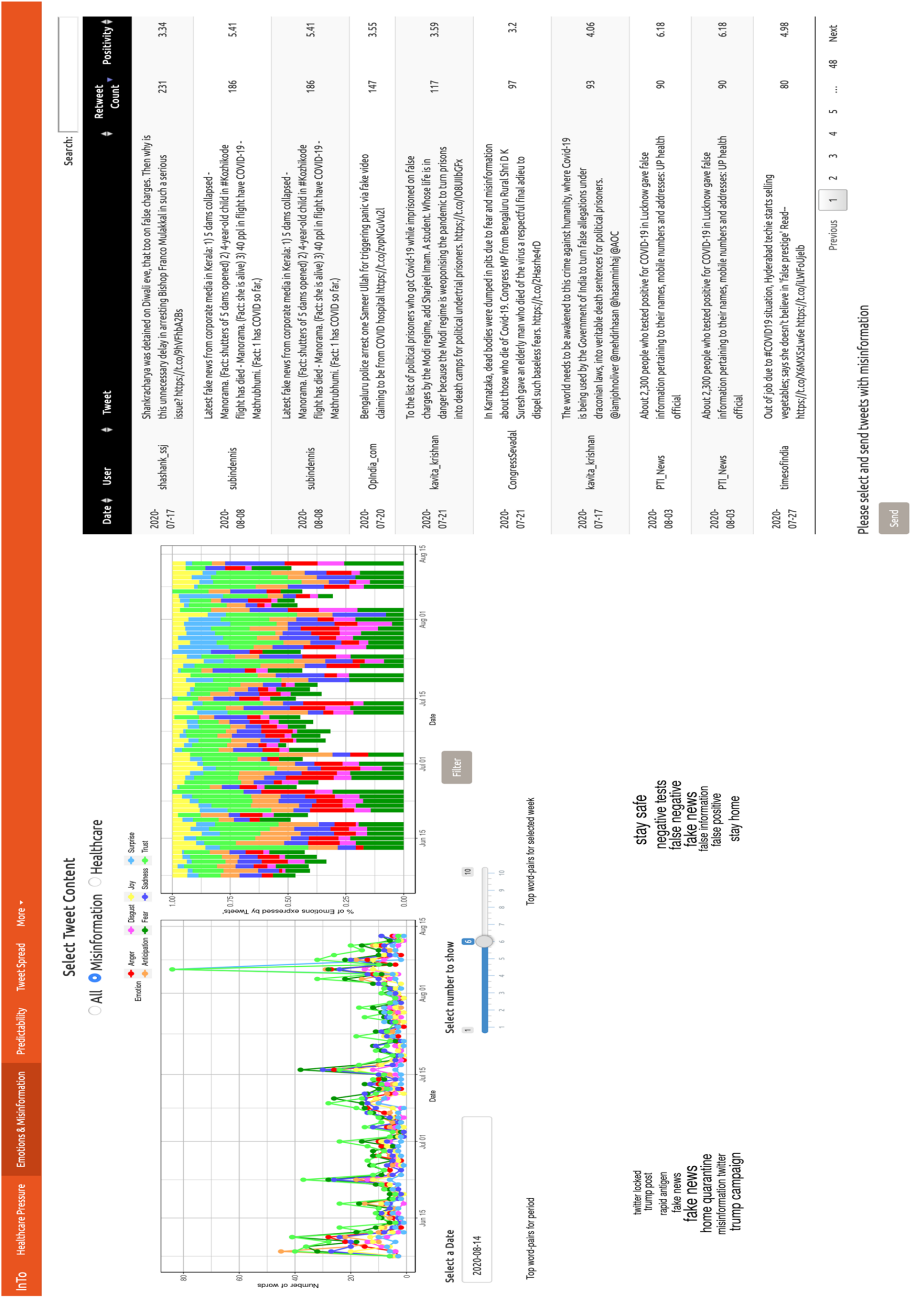
Dashboard Tab 2: emotions, top bigrams and tweets for predictive information, misinformation and healthcare. In the Emotions and Misinformation section, users are shown two time series of emotional affect, a table of tweet texts with their retweet counts and positivity, and a visualization of the top bigrams for a selected week. The time series on the left presents the absolute volume of emotional affects while the time series on the visualizes the proportional volume of each category. Users are given the opportunity to visualize the output for all tweets, or the subset of misinforming or healthcare tweets. They are also given the opportunity to select and send any tweet from the table to the administrators for inclusion in the misinformation subset of tweets. The administrators can redo the analysis of the impact and value of misinformation considering the newly identified misinforming tweets.

**Figure 5:**
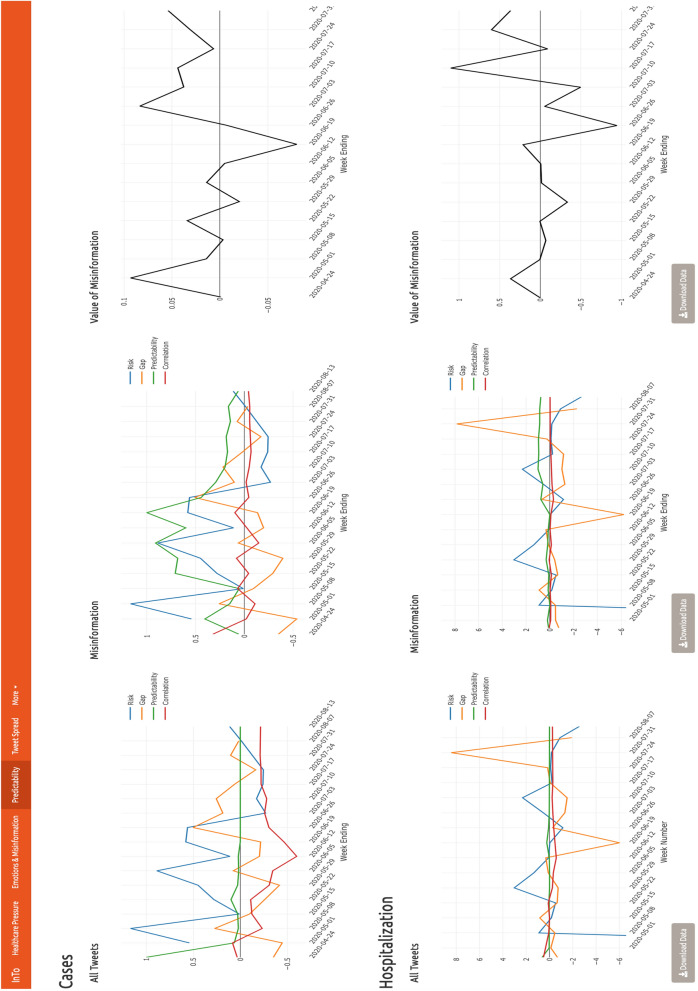
Dashboard Tab 3: predictability indicators. The Predictability section displays time series of risk, gap, predictability, and correlation indices along with the value of misinformation. In the left plots, users are shown these indices when the positivity of all tweets are used to forecast cases and hospitalizations. Middle plots show these indices when the positivity of the subset of misinforming tweets are used to forecast cases and hospitalizations. The right plots show times series of the value of misinformation (VoMi). For all plots, the x axis represents the time in weeks, while the y axis represents the value of the indicator as a percentage ratio. When users hover over a point, they are presented with the x–y coordinates.

**Figure 6:**
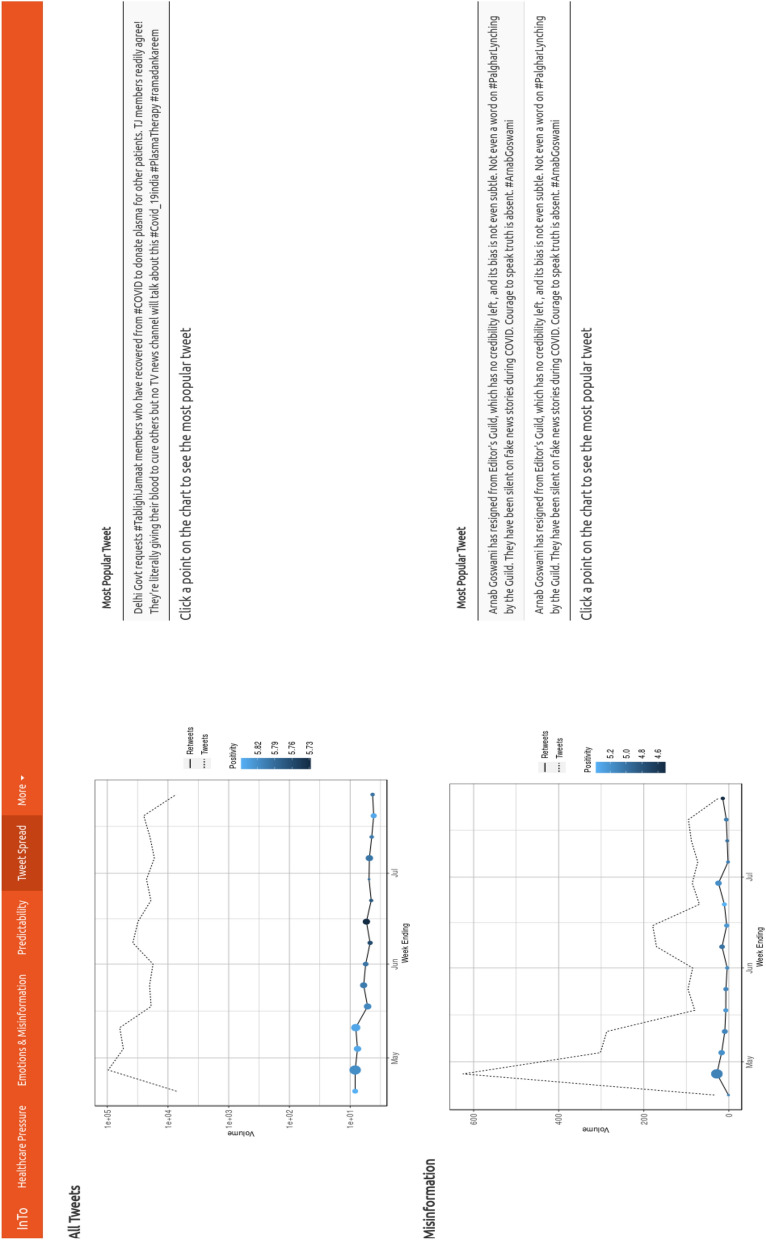
Dashboard Tab 3: information volume and spreading potential. The Tweet Spread section visualizes the spreading potential considering all tweets and the spreading potential from the subset of misinforming tweets. The x axis represents time in weeks while the y axis indicates the tweet volume observed in that week. The dashed lines shows the tweet volume for all tweets in that category while the solid line indicates the volume for the most retweeted tweets. The size of each point represents the mean retweet volume for that week, while the color represents the positivity of the most retweeted tweet observed the selected week. By hovering over a point the most retweeted tweet for that week is presented on the right.

Here we present InTo as an infoveillance system for the case of New Delhi and Mumbai during the COVID-19 pandemic between April and July 2020. New Delhi is chosen as the prototypical city to display because of its highly coupled social and epidemiological dynamics as empirically found from data (see Figs. 2, 3, 4, 5, 6). We included Mumbai to explore the external validity of our system when applied to a different city (see Supp. Figs. 1-4). In InTo, once the user selects their city of interest, results of analyses are displayed as a series of visualizations divided into four main sections corresponding to tabs of the dashboard: Healthcare Pressure, Emotions and Misinformation, Predictability and Tweet Spread (Figs. 2, 3, 4, 5, 6).

### Healthcare pressure

The layout and meaning of the Healthcare Pressure tab is displayed in Figs. 2 and 3, respectively. In Fig. 8 we present the results of using positivity from all tweets to forecast daily new hospitalization and daily new cases. Spatial forecasts related to misinformation are not shown spatially. The numbers displayed on the top of the dashboard refer to expected new hospitalization and hospitalization change from ARIMA (Eq. 5.3) for the entire city. Figure 3 illustrates, for New Delhi, how healthcare pressure $H_{P_i}$ can be interpreted as spatial gradients of hospitalization (meaningful of potential mobility gradients of people in need of hospitalization mediated by the presence of healthcare facilities), which is calculated as the difference between locally expected hospitalization and average hospitalization (E.q. 5.8). $H_{P_i}$ is visualized in a green-red color shade (where red is for the highest $H_{P_i}$) for *M* randomly generated points (10,000) over the city which are interpolated using the geokriging model (Eqs. 5.4–5.7) using the semivariogram of positivity. Positivity fluctuates around the same city-specific mean, while cumulative hospitalization grows exponentially over the course of the epidemic. Theoretical Gaussian and exponential variograms were the best fit for positivity and for cumulative hospitalization, as expected considering their time dynamics (left plots in the dashboard in Fig. 2). The area encompassed by each point is in the range 0.5–1.0 $km^2$, depending on the spacing between points; thus, our forecasts provide a high spatial resolution compared to other surveillance systems. The total number of hospitalizations in a selected area can be calculate as sum of new hospitalizations for all the points in that area. Figure S1 shows healthcare pressure for Mumbai.

For example, the user is shown that in New Delhi between April 15 and July 30 public positivity captured from COVID-19 related tweets ranged between 5.6 and 6.0 with a slight downtrend from 5.85 at the beginning of the period to 5.73 at the end. Meanwhile, there was a trend reversal in new cases and the cumulative hospitalization, with new hospitalizations showing an increase in the magnitude of fluctuations closer to the end of the period. Positivity was at its lowest in June when cumulative hospitalizations was at its highest but positivity was highest in July when hospitalization began to increase again. By using the linear relationship between hospitalization and positivity (see ARIMA model at Eq. 5.3), on July 30th we predicted the next week’s hospitalization to decrease by 381 hospitalizations (i.e. new hospitalizations displayed in the left plot of the dashboard), and cases to increase by 549. Cumulative hospitalization and cases were about 20,000 and 1500 on July 30th (left plots in the dashboard). Considering the spatial distribution of positivity and hospitalization in the two weeks before forecasting, via geostatistical kriging we forecasted two large clusters of hospitalization in the North-West and South-East and a smaller cluster in the center of New Delhi. In the high healthcare pressure areas colored in red, we estimated that there would be almost 200 new individuals in need of hospitalization (see color bar in the dashboard screen). The 200 newly predicted hospitalizations displayed in the dashboard constitute the peaks above the average (or the maximum healthcare pressure) in the entire city. The average is $\sim$12 according to the geokriging, and that corresponds to the ARIMA average shown on the top of the dashboard (see Fig. 2). The average new hospitalizations matches matches very closely the observed hospitalizations from surveillance (i.e. 11). Note that $\sim$200 hospitalizations are for few areas in the city and these extreme values are well above the average value for the period considered. For New Delhi, considering these results for the week displayed, hospital managers may wish to focus their attention to the North-West and South-East areas of New Delhi (lacking healthcare capacity as displayed by the geolocated and visualized hospitals in Fig. 2) where individuals in need of hospitalizations are potentially looking for treatment in other areas, and thus establishing hospitalization fluxes.

In Mumbai (Fig. S1), between mid-April and early-August 2020, tweet positivity from COVID-19 related tweets ranged between 5.6 and 6.1, but trended downwards over the period from 5.9 at the beginning to the 5.8 by the end. This downtrend occurred as new hospitalizations and cumulative cases increased, with large fluctuations in positivity coinciding with large fluctuations in new hospitalizations. Positivity was at its lowest in August when cumulative cases was at its highest, and new hospitalizations ended a downtrend and restarted increasing. We forecasted an increase of 140 new hospitalizations and over 2000 new cases for the week following August 8. At that time, cumulative hospitalizations and cases were 500 and 30,000 respectively. The spatial forecasts identified three clusters of cases hospitalizations located in the South, North, and East of Mumbai, with as many as 400 new individuals potentially in need of hospitalization. The cluster to the North appeared to have many more facilities to deal with the coming need than the cluster in the South and East (hospitals are indicated by a red H retrieved from a Google Map search of “hospital”). Consequently, managers could focus more resources to the north and east to maintain service levels.

### Emotions, top words, and misinformation

In the Emotions and Misinformation tab (Fig. 4), emotions—from emotion inference algorithms (see Sect. 5.3)—are extracted from the systemic information (all the tweets), misinformation-related tweets, and healthcare-specific tweets throughout the epidemic. Tweets for each category are reported on the right of the tab and some of these tweets can be directly reported to InTo as misinformation by social media (Twitter) users. Below we report results that can be inferred by using InTo, such as specific events, word pairs, users and associated emotions for New Delhi and Mumbai. These emotional categories were not included in the forecasting process, but assist users with interpreting high or low positivity scores.

In New Delhi, when considering all tweets, the dominant emotion over time was trust, followed by fear and anticipation; joy and sadness were the next most frequent; surprise and disgust were expressed the least. This distribution was observed for the subset of tweets related to misinformation as well; however there was one day, June 10th, when these tweets expressed more fear than they did trust. Tweet positivity was low on this day (see Fig. 2). As for tweets related to healthcare, trust was usually most expressed, but it was not as dominant as in the case of all tweets or misinformation. Furthermore, sadness seemed to be expressed much more among these tweets, especially in early June. June 10 was the saddest day considering healthcare tweets. On July 22nd, the most frequent pairs of words referenced were about “public health advice” and “self-quarantine at home”. A review of the raw tweets showed that many of the tweets were actually tweets of news articles made by organizations rather than individuals. Such tweets tended to be “neutral” in their positivity (i.e. centered around 5 without an increasing or decreasing trend), with values ranging between 4 and 6. This emphasizes the tendency of organizations, versus individuals, in manifesting risk-neutral perception patterns corresponding to average values of positivity.

In Mumbai (Fig. S2), the dominant emotion in the unfiltered set of tweets was trust, following by fear and anticipation; surprise was least abundant. A similar pattern was observed for tweets about healthcare, but the misinformation tweets appeared to harbor more sadness and fear. The top words in Mumbai referenced government advisories for social distancing and testing. Tweets with the lowest positivity were about a suicide believed to be associated with the societal stress (isolations, job losses, deaths, etc.) associated with the pandemic. Among the healthcare tweets were many messages related to increased testing at the facilities, but that those found positive were being sent home to quarantine nonetheless. The misinformation tweets largely contained accusations or identifications of false claims being spread on Twitter, and other social media platforms like Facebook, as yet unfulfilled promises of the government.

### Predictability and forecasting

Predictability indices (Sect. 5.5) are reported in the Predictability tab (Fig. 5) for both cases and hospitalizations as values over 100; yet, percentage changes are easily quantifiable. These metrics aid users in monitoring the accuracy of the model using all tweets and only misinformation-related tweets, as well as the value of misinformation-related tweets. The risk index in particular will show the same trend for the full tweet and misinformation-related datasets because it is based on the same data (the time series are reported twice to compare infection and hospitalization trends against systemic information and misinformation indices). A model-based risk indicator can be calculated to visualize the risk in terms of predicted values rather than data only.

For New Delhi, the risk index confirmed that cases were declining over time, despite momentary increases. Between May and June tweet positivity was under-predicting cases but then began to over-predict cases in July. Tweet positivity and cases showed a mostly moderately negative correlation (mean corr= −0.24). Although the value of the correlation was constant, suggesting a reliable model or stable dynamics, predictability was not stable until late June, when the predictability indicator became very small indicating lack of non-linearity, and thus implying high reliability in the linear forecasting of cases via the ARIMA model. All results suggest that tweet positivity from all downloaded tweets was most meaningful for forecasting the spatio-temporal spread in July, with relatively high uncertainty earlier. July has the highest correlation coefficient (in magnitude), lowest gap and non-linear predictability, as well as the lowest VoMi (Eq. 5.13). The subset of tweets related to misinformation showed a similarly negative though much weaker correlation with cases (mean corr = −0.01). This is concordant to the much higher non-linear predictability of misinformation manifesting the decreasing forecasting accuracy of ARIMA for this tweet subset. When considering all tweets, the model mostly under-predicted hospitalizations in New Delhi, with its largest under-prediction occurring in late June after hospitalization became the largest in the end of May (bottom left plot of Fig. 5). The largest over-prediction was observed in late July after hospitalization risk became very large. Yet, very large spikes in risk seemed to produce very large gaps in predictions. These large gaps are driven by misinformation as shown by the VoMi assessment that is higher at the end of the monitored period. The value of misinformation (Eq. 5.13) showed a gradual uptrend, indicating that tweets related to misinformation were decreasing the forecasting accuracy (based on linear correlation) of all tweets for cases and hospitalization as time progressed. Tweet positivity was mostly negatively correlated with hospitalization but predictability was low, especially for hospitalization.

For Mumbai (Fig. S3), the risk index displayed a decline in cases between the beginning and end of the period, while the hospitalization risk remained relatively stable, notwithstanding the decline closer to the end of the period. The accuracy of the model when predicting all cases trended upwards as indicated by the uptrend in the gap index, which varied around zero, slightly under-predicting cases. The predictability index when forecasting hospitalization was stable for the entirety of the series; it trended downwards until July, when it stabilized as well. Positivity was negatively correlated with cases (mean corr. $\sim$ −0.3). Using the positivity from all tweets tended to under-predict hospitalizations, despite two periods of relatively large over-predictions in May and July; the gap between predicted and observed hospitalizations was mostly close to zero. These large over-predictions coincided with spikes in the VoMI index, implicating the misinformation-related tweets as the source of this inaccuracy. Tweet positivity showed a slightly negative correlation with hospitalizations (mean corr. $\sim$ −0.01), with a predictability of index very close to zero indicating high linearity between positivity and hospitalizations.

The results from both cities underline the fact that there is more linearity between new hospitalization and positivity than cases and positivity, such that the ARIMA forecasts are more reliable for new hospitalization. Despite this average result we observe that larger fluctuations in indicators are seen for hospitalization than cases, likely underlying the necessity to include other predictors for extreme hospitalization events. Lastly, time series of indicators for all tweets and misinformative tweets are quite similar due to the low detection of misinformation; nonetheless time-point values are different as manifested by VoMi because misinformation, although small, exist and impact forecasts.

### Tweet spread

The Tweet Spread tab (Fig. 6) shows the volume of tweets and retweets, as well as their positivity, for the systemic information and misinformation set. Users are also able to identify the most popular tweet from the full set and the misinformation-related subset.

There were between 10,000 and 100,000 tweets per week related to COVID-19 in New Delhi. The volume of retweets was much lower in comparison, not exceeding 10 retweets, and had positivity values approach 6 meaning they were more positive than the average neutral value of 5. Additionally, both tweet volume, retweets and positivity are slowly decreasing over time which manifest the lower COVID information production and decreasing positivity. The number of misinformation-related tweets was the highest in the early days of the pandemic descending relatively rapidly as time progressed. The retweet volume was very low compared to the full tweet set (the difference is about three orders of magnitude) and most of the popular misinformative tweets had low positivity.

From Mumbai (Fig. S4) we observed between 1000 and 100,000 COVID-19 related tweets per weeks, with less than 10 retweets per week measuring between 5.7 and 5.9 positivity. The misinformation set contained between 10 and 400 tweets per week, with less than 5 retweets per week of positivity 5.0 to 5.4. The number of misinformation related tweets was highest in May and mid-June.

The most popular misinformation-related tweets underline the fact that misinformation is not necessarily carrying deceiving information but also information about perceived wrong behavior in populations. Thus, misinformation can capture more the dichotomy between common and divergent groups in the area analyzed. Additionally, the large difference in volume of all tweets ($\sim 10^5$) and misinformative tweets (that are less than $10^3$, two orders of magnitude less than all Tweets) explains why time series dynamics of predictability indicators for the systemic information and misinformation predictors (Fig. 5) is very similar but time point values are different.

### Model calibration and validation

**Figure 7:**
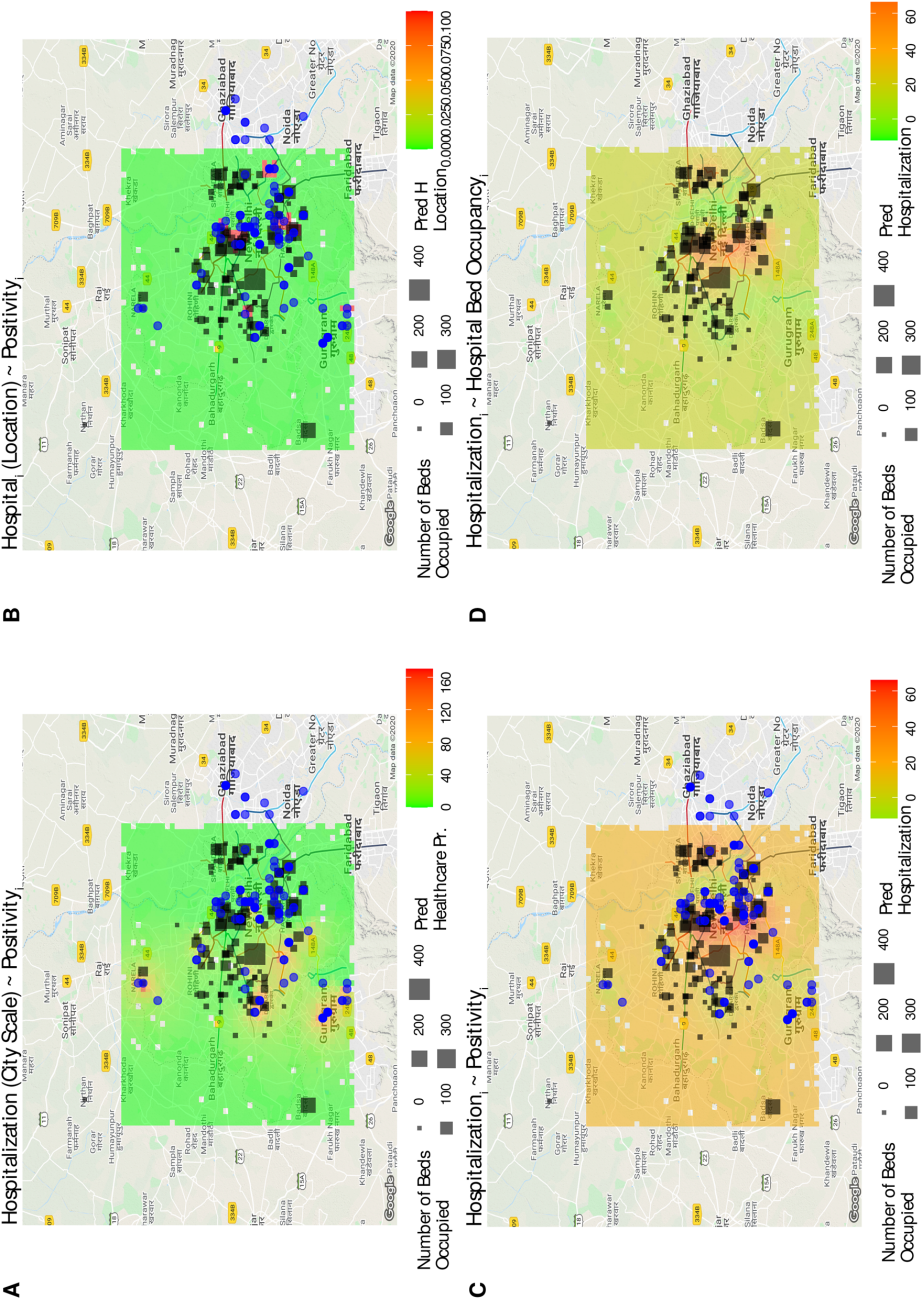
Spatial validation of geokriging predictions of cumulative hospitalization. Predicted and observed cumulative hospitalization ($\hat{H_T}$ and $H_T$) are calculated as a function of spatially explicit positivity $P_i$ and hospital reported hospitalization (plot A and C) via geokriging. Plot A shows the prediction offered by the dashboard where spatial healthcare pressure $HP_i = \hat{H_{T_i}} - H_T$ is determined as difference between local and total hospitalization at the city scale. Plot B and D report predictions of hospital location based on positivity and cumulative hospitalization based on reported bed occupancy only, respectively. The relationship on the top of each plot is reporting what is used in the geokriging calibration, while what is predicted is reported at the bottom. Squares indicate officially reported hospitals designated for COVID-19 patients, while blue points indicate geo-located Tweets. Predictions are for the period 21 July–11 August 2020.

Results of the model validation over space (for the optimal predictor set) are displayed in Fig. 7. Plot A shows the forecast of spatial hospitalization based on geospatial tweet positivity and city scale hospitalization. Predicted hospitalization based on Tweet positivity suggested there would be high hospitalization pressure $H_P$ (Eq. 5.8) in areas, such as Narella, Gurugram and Dwarka (SW part of the city), which were unaccounted for by the monitoring system just focused on bed occupancy (and yet on models based on that occupancy shown in plot C and D). The highest peak of $H_P$ is 160 and the average of healthcare pressure over space is very close to the average of hospitalization at the city scale. However, the geographical distribution of healthcare pressure is different from the distribution of hospitals because geokriging is extending spatially the positivity-hospitalization relationship (that shows an inverse proportionality between these variables) that is beyond hospital locations. Nonetheless, tweet locations highly predict hospital locations as binary variables (Fig. 7B).

When performing interpolation via geokriging based on hospital-scale data alone (Fig. 7D), high hospitalization was predicted in the center of the city, with gradients of hospitalization decreasing outwards. This predicted hospitalization reflects ($\sim 80\%$) the distribution of bed occupancy as expected. The predicted hospitalization considering hospital-scale occupancy and positivity (Fig. 7C) matches $85\%$ the hospitalization based on hospital data only (Fig. 7D). The former is however predicting higher hospitalization in other areas beyond hospital areas, and this emphasizes the fact that the model is also predicting healthcare pressure as individuals likely in need of hospitalization. Note that the range of hospitalization for predictions of plots C and D in Fig. 7 are the same with maximum cumulative hospitalization equal to $\sim$65 for the period 21 July–11 August 2020.

**Figure 8:**
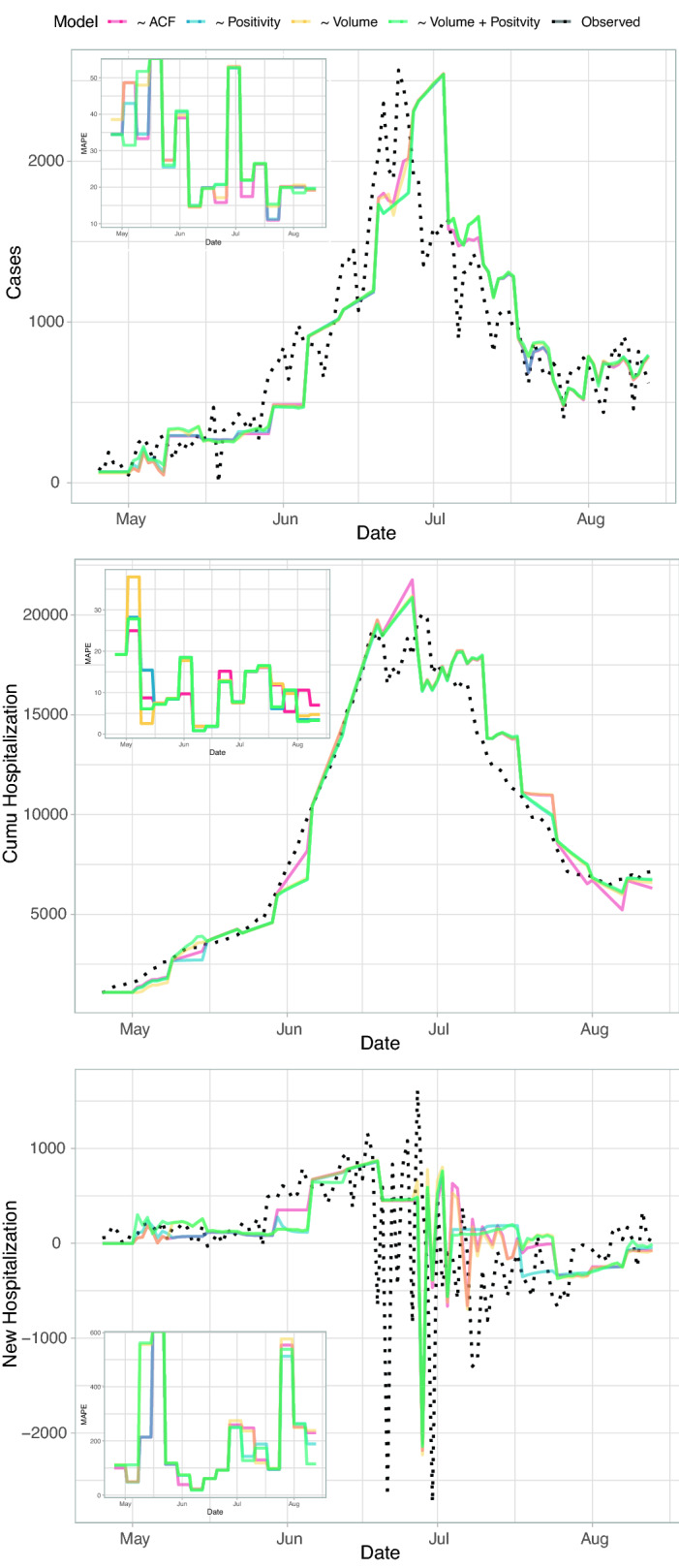
Hospitalization and case forecasting for different predictive models. The results of ARIMA forecasts with different models in terms of predictors are shown for cases, cumulative and new hospitalization (top to bottom) for New Delhi. ACF is ARIMA based on epidemiological data only, while all other ARIMA models are based on positivity, Tweet volume, Tweet volume and positivity combined (red, blue, yellow, and green curves). Black dots are from observations at the city scale. All curves are at the daily resolution.

Figure 8 shows the calibration and validation of the ARIMA model which is useful for selecting the optimal set of predictors. The results of ARIMA forecasts with different models in terms of predictors are shown for cases, cumulative and new hospitalizations for New Delhi. ACF is ARIMA based on epidemiological data only, while all other ARIMA models are based on positivity, Tweet volume, Tweet volume and positivity combined. The model that minimizes the mean absolute percentage error (MAPE, in insets) is based on positivity only because of its highest predictive power for fluctuations in healthcare pressure (cases and hospitalization). However, the model with volume and positivity has similar MAPE because of the ability of volume to predict the largest extreme variations in hospitalization. MAPE is larger for new hospitalization than cumulative hospitalizations due to the larger stochasticity of the former than the latter over time. The departure of forecasted values from observations is the gap index in the dashboard (Fig. 5).

The (*p*, *d*, *q*) parameters of the ARIMA model (Sect. 5.4.1) manifesting seasonality, memory and fluctuations are on average [0, 1, 1] for all models including ACF, [0, 1, 2] toward the end of the monitored period that highlights the increase importance of fluctuations, and [1, 1, 2] for volume and positivity that highlights the higher seasonality of tweet volume and ability to capture larger extremes. (*p*, *d*, *q*) parameters increase if misinformation is used when predicting hospitalization and cases, and this is in synchrony with our findings that evidence how non-linear predictability (via TE) increases because of the higher memory due to long-range time-delayed effects of misinformation. Average results of social and epidemiological variables for New Delhi and Mumbai are in Table 1 considering different city areas and time periods.

**Table 1:** Average socio-epidemiological values for the New Delhi and Mumbai. Average weekly values for hospitalization *H*, cases *I*, tweet volume, retweet and positivity (*V*, *R*, and *P*), as well as Pearson correlation between positivity and hospitalization. Average *VoMi* also provided. The Pearson correlation is proportional to the first regression coefficient of the ARIMA forecasting model and the geokriging factor of hospitalization predictions. The higher $\text{ corr }(P,H)$ the higher the potential risk aversion for the city (areas and time periods) considered. It is empirically observed that the higher the risk aversion the lower the social (Twitter) generation of information and the healthcare pressure defined by combined case and hospitalization magnitude. VoMi is expected to be higher for less risk-averting city areas (and time periods) with higher incidence (thus misinformation is more predictive of cases and hospitalization) and these areas/time periods should appear more local in terms of circulating information.

City	${\bar{H}}_t$	${\bar{I}}_t$	${\bar{V}}_t$	${\bar{R}}_t$	${\bar{P}}_t$	corr(*P*, *H*)	*VoMi*
New Delhi	100	1000	10,600	9	5.75	$-0.5$	0.00
Mumbai	500	1250	10,500	10	5.85	$-0.1$	0.10

## Discussion

We have demonstrated the use of InTo to calculate tweet positivity to forecast and predict the spatio-temporal spread of COVID-19 healthcare pressure. However, the model can be applied to any disease or public health phenomena of interest via properly tuning the forecasting models. In New Delhi we inferred that the population was relatively positive in the messaging, expressing mostly trust, despite the high case load and hospitalization. This weak negative correlation manifesting risk aversion—due to the expected decrease in positivity for increases in hospitalization—was statistically useful for predictability purposes considering both geostatistical kriging and ARIMA models that use correlation values (Eqs. 5.3. and 5.7).

We showed that hospitalizations could be expected to concentrate in certain areas of the city, suggesting those clusters to be the focus of additional public health surveillance and healthcare resources since new hospitalizations may occur. We found that misinformation does affect the accuracy of the model and provides another illustration of the impact of misinformation: it can impact even our ability to properly forecast healthcare pressure but not necessarily negatively (in terms of reduction of prediction accuracy) throughout the pandemic. This impact was found to be positive, yet improving prediction accuracy, at the beginning of the epidemic (despite the higher volume of misinformation) and negative at the end of the epidemic likely because the delayed effect of misinformation spreading.

### Data uncertainty and model transferability

The success of any infoveillance tools rests also on the availability of data. Better quality data can likely support more accurate and more meaningful forecasts. Better data refers not only to the representativeness of the data but also to the granularity and compatibility of the data as well in relation to what is predicted. In terms of granularity, this could be hospital level rather than state or national level hospitalization data for example. We showed in Fig. 7 that the geostatistical kriging model performs much better—in terms of predicted hospitalization—when spatially explicit hospital data are provided, particularly when the objective is also to capture reported bed occupancy rather than average expected hospitalization at the city scale solely. Compatibility would mean not only using universally accepted terminology, but formatting the data in the same way to ease data processing. Certainly a huge discrepancy exist between social and epidemiological data (considering spatial and temporal resolutions as well as data volume), and then data processing becomes a time consuming process potentially carrying systematic uncertainties. Technology exists to translate data which is formatted differently, but it remains important that data stewards communicate with epidemiologists, “infodemiologists” and decision makers to determine a usable design. This is particularly important in the context of pandemics and emerging infectious diseases although localized.

Our concern is directed more towards epidemiological data rather than social media data, at least in terms of predicted patterns, i.e. temporal dynamics of cases and hospitalization. Social media users generate terabytes of data and many platforms have policies that allow restricted access to data, especially for academic purposes or some other public good purpose. Additionally, social information is country specific, for instance dependent on available and popular social media as well as local language, and yet it is much more ”subjective” and with a very high degree of uncertainty. Vice versa, despite epidemiological data has proven to be more difficult to collect and share, they are more objective data to compare among countries since one case or one hospitalization is one incidence unit everywhere. Officials must also decide on what data is important to collect or monitor, as there are several epidemiological metrics that are important or valuable for different reasons. For example, whereas a hospital manager may find hospitalizations or cases most relevant, a public health official may wish to focus on the ratio of deaths to cases. Our tool can be designed to accommodate as many metrics as are deemed relevant, although the predictability of these additional metrics would first need to be established (see Sect. 3.3 for more on this). Beyond these aspects, we emphasize that it would take effective coordination as hospital managers and public health officials collate and share data via application programming interfaces (API) for highest efficiency and timeliness in generating results.

The paper methodology is universally applicable to any geographical area of interest at any desired scale (e.g. from cities, regions and countries) and independently of administrative boundaries. We point out that the mathematical and computational model infer distinct patterns (in the form of case-positivity and hospitalization-positivity patterns) potentially underpinning social patterns in terms of risk perception and information flow that are highly linked to each other^30,31^. This is evident considering the case of New Delhi and Mumbai. This type of modeling, focused on pattern inference, has been widely adopted in many areas of science, particularly when using probabilistic approaches (such as statistical physics and information-theoretic ones; see Li and Convertino^28^) that are not tight to specific socio-ecological processes but characterizing propagation of probability distribution functions (or their statistical moments) in order to capture macro-features or mechanisms. For instance, see Convertino et al.^32^ in the context of Leptospirosis to link epidemiological and environmental dynamical patterns. Recently, in relation to COVID-19 Chan et al.^33^ inferred patterns of intervention effectiveness from incidence curves over time and portfolio sets of other interventions: risk communication was found as the most important intervention independently of the media used in spreading risk information as well as other country specific social features. This stresses even more the applicability of our model (related to spread information) and the findings of macro-risk perception patterns, by keeping in mind that these patterns are bounded by the social media used. Certainly, another aspect is related to how much social information is revealing realistic risk perceptions but that is another issue related to representativeness of social information that requires further investigations.

### Population representativeness of socio-epidemiological data

An issue connected with data availability is the matter of representation, that is, the extent to which the data include enough heterogeneity to reflect the complexity of the population for which the data set is assembled. This is particularly relevant to social media data such as Twitter data. The demographics of users can differ significantly by biology, socio-cultural and economic class, location and the availability of technological infrastructure^34,35,36,37^ so individual/community experiences and perspectives can differ from the wider population^38^. Even the choice of language might limit the representativeness of data used in the model: InTo currently uses English, which is spoken in India, but not by a majority. One also has to consider the inclusivity of the search term. Our use of ’OR’ instead of ’AND’ made our search more inclusive rather than restrictive thereby increasing the potential volume of tweets returned. Other choices would have certainly provided other predictability indices; and then one of the future improvements would be extracting the set of constraining hashtags that maximize predictions overall among all possible choices of hashtags. However, this choice would require a much higher computational cost and, in addition, fitting data the closest (versus providing the full range of feasible predictions in a Maximum Entropy perspective) is not always the optimal choice due to the presence of systematic uncertainty in data. Therefore, our current InTo version is not necessarily bounding the model-data gap considering all feasible factors (from language to hashtags), nor a fully causal investigation, but a model defining the simplest and most informative inputs and outputs to represent dynamics of population patterns. Further work will define more clearly importance of underlying factors and the absolutely optimal model form.

Tweets in a city contain information of spatially separated events about the same process; thus spatial spread of COVID and top tweeted pairs can be calculated over geolocated Tweets. Posting time and content (related to volume and positivity) is very weakly dependent on the social media platform. Additionally, social media users tend to interact outside of their usual social networks or real-world socio-economic class much more on these platforms^36^, creating opportunities for groups absent from these platforms to be heard in a latent way. Furthermore, tweets report information that may not be reported by official media and/or that may circulate in real life events (e.g. just spoken information). This is also the reason for which InTo can be used by users as a reporting information/misinformation tool via registering their Twitter account. We suggest this “Digital Health” feature particularly relevant for healthcare workers.

Twitter penetration can differ between and within countries, but tweets still show high relevance for predicting spatio-temporal patterns of infections and hospitalization. Additionally, emotional affects are highly linked to local non-Twitter media and languages, as we see high volumetric correlation with local newspapers articles and retweets of English tweets in local languages. Certainly, demographic and other features of the tweeting population are relevant for how the virus spread but not the whole complexity is needed for forecasting purposes in the short and long term. Nonetheless, this version of InTo is a proof of concept version and will likely investigate and include other social media platforms, languages, information features, visualization options, diseases and socio-environmental phenomena in future versions for investigating processes and practical applications.

The model is certainly sensitive to the choice of the social media considered and that is also a country-specific factor. Thus, in principle, one should use the most popular social media in the country analyzed in order to gather the highest resolution social information to characterize risk perception patterns. However, in terms of predictions, predictive accuracy is not necessarily related to the most popular social media because even a smaller volume of information can maximize prediction accuracy. A distinction should be made between predictive patterns versus patterns reflecting real processes. For instance, for the country analyzed (India) the predictive accuracy is relatively high ($\sim$60$\%$ of hospitalizations). As for realistic risk perception patterns it makes sense to discuss about how much one social media is representative of the whole population rather than what is proportion of users in one social media, since volume of users does not necessarily correlate with representativity. For instance, Twitter users may report the vast majority of events occurring in a population, considering also retweets of local newspapers in local languages. Just for statistical information we report that Twitter (during the study period, i.e., April–July 2020) is used by 6$\%$ of the population in India (source https://gs.statcounter.com/social-media-stats/all/India). It should be noted that these penetration rates are relative to each country’s total population; in a global perspective India is the 3rd largest countries in terms of Twitter users (https://www.statista.com/statistics/242606/number-of-active-twitter-users-in-selected-countries/). Further studies are however necessary to understand the variability and representativeness of positivity across social media and its relationship with usage also for certain social demographies. An important information non-linearity that should be considered when establishing effective representativeness is also: (i) the interdependence of Twitter with other social media (where Tweets carry information of these media; e.g. in India Tweets can be found or relate to Facebook, WhatsApp, Instagram, YouTube, Snapchat, Twitter, LinkedIn, and Quora information that are the other social media in terms of usage); and (ii) geographical dependencies related to users in a country that are connected to many other countries’ users, and yet strongly influenced by other countries’ social media production.

### Predictive causality versus forecasting, and non-linearity

Even when considering the issues of data availability and representativeness, the advantage of InTo is that it focuses on patterns rather than causation. InTo does not purport to have found nor to be exploiting a causal relationship between tweet positivity and healthcare pressure. Rather, it exploits spatio-temporal patterns and correlations that might not be physically significant (although arguable in an information dynamic sense), but that are nonetheless practically useful probabilistically. The relationship between sentiments and behaviors are quite complex, and there are many other variables in the complex reality of phenomena considered that are however not all needed when forecasting population outcomes. There are population factors such as sex, socio-economic status, proximity to affordable healthcare facilities and the availability of insurance or some other means of paying that certainly impact real processes of individuals. There may even be socio-political realities at play that force individual behavior. However, the key goal of InTo—in a complex system science purview—is the prediction of population patterns considering the most essential predictors without making any assumption on the underlying processes. Complicating the model comes at a cost, not just in the acquisition of data—because such data may not be available or costly to acquire—but also in the applicability of the resultant model that would be highly sensitive, extremely hard to calibrate and full of unchartable uncertainties. A model that enables reliable forecasts with a reasonable level of accuracy given a variety of scenarios should be the aim of any information system model.

In InTo a forecast refers to the estimation of future outcomes (in short term) which uses data from previous outcomes, combined with recent or future trends. Forecasts like those from the application of ARIMA models imply time series and future point estimates, while predictions do not. A prediction is based on probabilistic patterns (e.g. probability distributions, trends, and total uncertainty reductions) and yet of “possible outcomes” in the long-term. This is the case of geokriging and the pattern that can be obtained by using the predictability indicator (Eq. 5.12). Forecasting does not imply predictability nor the contrary, but in principle, optimized forecasting implies strong predictability for the whole time period considered. Vice versa, predictability of patterns does not guarantee the ability to have highly accurate time point estimates. InTo is providing both in order to support public health in almost real-time decision making and long term sensitivity of social surveillance for epidemiological outcomes.

The accuracy of this system must be monitored if it is to be trusted to inform meaningful public health measures. Although the general form of the model as described in Eqs. 5.3–5.8 remains the same, additional parameters, such as p, d, and q for the ARIMA model, were allowed to vary. Also, as the entire history of data is used for forecasting, an ever-increasing data set is available for training which provides more from which to learn. For example, our system applies the ARIMA model in an evolutionary way rather than as a static model: as new data is added, the ARIMA model is recalibrated considering the extended data. This reduces (yet does not eliminate) concerns like overfitting, which would be more problematic if we used an unchanging model imputed from an immutable training set. Furthermore, our model does not attempt to make forecasts for values too far out-of-sample: we make predictions for a single week ahead as longer horizons typically reduces the accuracy of models. Notwithstanding this, our inclusion of the Gap Index in the Predictability tab provides sufficient caution to the user: as the Gap index increases, users are alerted to potential issues with the model as designed.

Social stress certainly impacts epidemiological dynamics (as widely reported, e.g. see^39^ and Campo-Arias and De Mendieta^40^) but this aspect was not analyzed in our research. Social stress can be considered as a population-level factor inducing changes of positivity and social media production over time after prolonged hazard exposure (in this case the COVID-19 epidemic and controls). Yet, social-stress, likely measurable by consistent decrease in positivity, may lead to non-linearity such as time-delayed changes in hospitalization. In Kastalskiy et al.^24^ a model for the COVID-19 epidemic was proposed by combining the dynamics of social stress (as sociophysical phenomenon in the form of alarm-ignorance-resistance-exhaustion dynamics reflecting populations’ adaptation syndrome) with a classical susceptible-infected-recovered “SIR” epidemic model, where the susceptibles are split into three social-stress groups. This integrated model described with high accuracy the available epidemiological data for 13 countries and highlighted the country-dependent non-linear dynamics (driven by social vs. biological dynamics of the virus) for the whole period considering overall temporal trends and distribution. However, we emphasize that non-linear dynamics of processes does not imply non-linear patterns and patterns are scale-dependent. For instance, in our study at the weekly scale we do not observe non-linearity in the socio-epidemiological relationships (fitted by the ARIMA model), despite real processes are obviously non-linear, but these relationships and their probability distributions over longer time-scales than a week are non-linear and non-normal, respectively. Thus, a critical distinction should always be made between patterns and processes and models are tendentially always pattern-oriented tools even when discretize analytically some selected mechanisms under hypothesized assumptions^41,42^.

### Value of misinformation

Identifying misinformation is a chief concern in infodemiology via infoveillance, not to mention in other areas of society like sociology and politics. Methods that use the probabilistic and lexical features of text in order to determine whether they represent misinformation^43^ abound. These methods depend on datasets that contain messages which have already been labelled misinformation by experts a priori. Keyword-based strategies, as we employed, are problematic^44^ so it would be more accurate to describe our results as the value of the topic “misinformation” rather the value of specific misinforming messages. This notwithstanding, we recommend validating the outcome of any keyword to ensure that the value of the proper messages are being evaluated (e.g. truly misinforming messages rather than accusations misinformation). The set of misinforming messages considered by InTo includes tweets already directly labelled as or questioned to be misinformation by users, having most likely already gone through a vetting process. The advantage of this approach is the use of a human- and crowd-based classification which overcomes the challenges of assumption-driven lexical analysis by model. Interestingly, a posteriori we confirmed (via reviewing Tweets one by one and considering their incorrect or false information) that the vast majority ($\sim 95 \%$) of misinformative tweets are truly misinformation and this misinformation set showed much larger dissimilarity—in terms of word diversity, volume divergence and asynchronicity—with respect to cases and hospitalization than the full tweet set. This emphasizes how dynamical properties of information are essential in categorizing different types of information, as well as how crowd-based self-reporting is relevant. In the literature there are still some debates about this topic but those seem platform dependent. For example, Jiang, S. and Wilson, C.^45^ suggested that user comments do not provide sufficient predictive power when attempting to classify misinformation, but a recent study (see Serrano et al.^46^) successfully utilized user comments on YouTube videos instead of parsing these videos to classify misinformation with high accuracy. Nonetheless, our attempt at measuring the value of these messages exemplifies another useful and customizable feature of our system. For example, a user may be interested in the value of other topics, such as vaccines. Future versions of this system can enable users to measure the value of any topic or a set of topics that accompany their disease of interest. Further research may detect keywords in an autonomous in term of their salience for the investigated topic and/or for increasing prediction accuracy.

Our results found that misinformation-related tweets provided at times more time-point accurate forecasts of healthcare pressure than forecasts based on all tweets. We observe that misinformation positivity shifts the forecast error based on all tweets to higher positive values (implying positive VoMi); yet, misinformation is slightly contributing to overprediction but considering its magnitude this overprediction is positive in consideration of surveillance underreporting and other systematic errors. This is not to say that misinformation is good in an absolute sense; in fact, it remains important that accurate facts are disseminated to people as the consequence of acting on incorrect information could imply wrong behavior leading to higher cases and hospitalization. Rather these findings show that misinformation—in its positivity rather than volume or messages—is useful for forecasting. This is related to the use of positivity as a novel aspect in characterizing social media content and to the fact that positivity fluctuations of quickly generated misinformation tend to have long-term consequences on the predictability of the unfolding epidemic (misinformation that of course can have impact on the social behavior of populations). This is manifested for instance by a higher predictability indicator of misinformation (Fig. 5) as well as the higher (*p*, *d*, *q*) parameters of the ARIMA model (Sect. 5.4.1). Additionally, the full tweet information may contain too much “entropy” of messages that do not quite reflect people sentiments about the epidemic despite not being misinformation. Thus, public health organization could use positivity embedded in misinformation to protect the public, and then seek to eradicate.

### Social value of InTo

The most immediate value to society of InTo is through appropriate social media signal monitoring and by complementing traditional epidemiological surveillance which allows optimal healthcare planning during public health crises. As a novel and innovative infoveillance cyberinfrastructure (because available online and systematized in its function), apart from monitoring the spread of social chatter, InTo enables the public health system to properly plan for inevitable fluxes of people in need of care.

Public health officials and healthcare institutions need a way to cost-effectively determine whether they are able to meet the impending healthcare demands via considering both information and disease epidemics that we showed to be non-trivially and strongly coupled. Additionally, InTo enables public health officials to evaluate customer satisfaction of the healthcare system during the epidemic/pandemic. This is performed by evaluating sentiments of words related to healthcare in terms of emotions, positivity and specific content of social chatter. Content that can point out specific hospitals, physicians and treatments, as well as users. Thus, individuals are able to review what the general public posts as problems on social media about the local healthcare infrastructure and global issues. Also, information about which institutions are operating beyond their capacity, and what particular department may be operating poorly or successfully is available. Yet, InTo responds the need of predictive, personalized and precise health in an unprecedented way by both capturing information-driven salient population patterns and individual needs.

By monitoring public expressions, InTo provides some insights into emotional affects of the population in response to disease spread. This can also illuminate the importance of psychological states in response to these crises, which may be precursors to post traumatic stress disorders (PTSD). Other studies^47,48^ showed how word choices reflect mental health states in long term and these may be predicted by performing a systemic functional network analysis of the tweet text extracted by InTo. This would also further link latent social and epidemiological outcomes explicitly.

Finally, InTo enables to monitor the spread of misinformation during public health and social crises, as well as evaluate the impact of any intervention, in the form of risk communication, they enact. InTo provides volumetric measures of misinformation generation on social media over time and geographical domain, as well as quantifies how misinformation affects forecasts of case and hospitalization (i.e. VoMI) that potentially relate to real-world misbehavior dependent on circulating misinformation. Therefore, the performance of interventions against misinformation can be measured by the volume of misinformation that is reduced as well as by the uncertainty reduction in forecasts. In this sense, InTo provides an extra evaluation of the surveillance system by considering misinformation as extra uncertainty or uncertainty reduction, depending on its negative or positive impact, on prediction accuracy. Comparison of multiple information sources and model predictions across multiple criteria over time time, is a rigorous and efficient way to evaluate surveillance systems and likely detect the most reliable source of data^20^.

## Conclusions

Infodemic Tomography (InTo) is proposed as a cybertechnology to monitor and visualize the spatio-temporal co-causal variability of social media positivity and healthcare pressure (as cases, hospitalization and misinformation separately) during epidemics and public health crises. The most salient points to mention about InTo are listed below.

- A clear linkage between epidemiological and information dynamics (in terms of positivity) is detected via linear and non-linear patterns, inferred through via linear regression and transfer entropy models, respectively—that are potentially revealing risk perception and information randomness in populations. These patterns are useful for predictions of epidemic dynamics, complementing traditional surveillance, and analyses of social media dynamics (generation, absorption, spreading, diversity and positivity) that have the potential to design risk communication strategies which aim to enhance or correct information shared in the target populations. Combined socio-epidemiological patterns can reveal risk perception patterns. For instance, Mumbai and New Delhi are shown to have the lowest and highest potential risk aversion considering the average positivity-hospitalization correlation (that is negative in sign, where, vice versa, a positive correlation would have implied risk seeking behavior).
- Location of tweets is deemed relevant to predict hospitalization where it is officially reported (interestingly, $\sim$60$\%$ of predictions of hospitalizations coincide with the reported total bed occupancy (in the test cities of New Delhi and Mumbai) and in locations where people are potentially in need of hospitalization. Yet, geospatial tweets (and associated positivity) are convenient transfer functions of epidemiological information to small space-time scales and inform about potential fluxes of healthcare demand that are useful for dynamic healthcare management. Forecasts of cases and hospitalization are provided at very high resolution ($\sim$ $m^2$) one week in advance by using a linearized ARIMA model. Risk and gap indicators are provided to measure the trend and model-gap difference of the epidemic weekly. A predictability indicator (normalized transfer entropy TE over the maximum TE across time) is developed to monitor the uncertainty reduction of Twitter positivity for epidemiological dynamics, thus to test the non-linear predictive causality in contrast to the linear forecasting of the ARIMA model. The lower TE the higher the forecasting accuracy due to the low non-linearity between positivity and cases.
- Misinformation is extracted by directly mining population-reported misinformation (via misinformation-related hashtags) and can be tested a posteriori via manual classification with public health officers cooperation and automated model-driven testing of dissimilarity (divergence, asynchronicity and diversity) from the systemic COVID-19 information over time. The Value of Misinformation (VoMi) is introduced as the impact on forecast accuracy calculated as the difference of gap indices (potentially negative over time) for the systemic and misinformation datasets. VoMi trends are city-specific and negative if they are increasing over time because they imply high impact of misinformation on short-term forecasting. VoMi is typically low or negative because it is highly non-linear, and yet not very informative for forecasting sudden events; however, it carries higher predictability (as uncertainty reduction) for delayed long-term extremes and probabilistic patterns as shown by high values of transfer entropy.

In conclusion, InTo encapsulates the future of public health management with the the fusion of multiple surveillance streams: from traditional epidemiological and healthcare data to model-inferred social sentiment data. As technology develops and the public creates and consumes information via internet, epidemiology will need to consider the spread of social information not only as a problematic element but as a solution for disease tracking and optimal risk communication. For instance, ad-hoc social messages by authorities can counteract misinformation that is sensed online, as well as social media inferred cases (or model predicted) can complement traditional public health surveillance. InTo shows that sentiments from digital messages can forecast the incidence and spread of healthcare pressure for areas besieged by a public health crisis. In terms of forecast, it is near-real time, accurate, reasonably inexpensive and easy to use in a computational sense. Infoveillance tools like InTo can only get better with higher quality data from traditional surveillance systems on which validation should be performed, but more importantly with the collaboration between developers and stakeholders to effectively create solutions that are useful for effective decision and policy making. Future work will potentially entail expanding social media platforms and diseases to be monitored. Other validation experiments to improve InTo accuracy and utility are needed in data-rich areas. Via collaborations with public health officers, stakeholders and volunteers with interests in social computing we will seek for releasing InTo as a globally implemented cyberinfrastructure for public health research and practice.

## Material, methods and implementation

### Twitter data mining and preprocessing

Data collection occurred weekly beginning in April 2020. Only English language tweets within a geographical bounding box (reflecting the target geographical area of the city considered) were retrieved from Twitter using the rtweet package^49^. The choice of English was dictated by the lack of robust computational tools usable for other language translations (also considering the big-data size of tweets) and the complexity of the languages for the country considered (i.e., Hindi and Marathi for New Delhi and Mumbai, respectively); the latter would make the uncertainty in positivity scoring of words very high.

Search terms are hashtags that were identified given their rank on a list of the most popular Twitter terms on a daily and weekly scale (the search was done by comparing https://getdaytrends.com/ and https://trends24.in/). Our search query for the COVID *systemic information* was constrained to the hashtags “covid OR coronavirus OR quarantine OR stay home OR hospital OR covid OR covid19 OR covid-19 OR coronavirus OR quarantine OR stayhome OR hospital”. Thus, we downloaded close to 30,000 tweets daily between April 15 and July 30, 2020 for New Delhi (defined as “National Capital Territory of Delhi” by Twitter in the box $28^{\circ }41'25.9''N, 76^{\circ }83'80.7''E$ to $28^{\circ }88'13.4''N, 77^{\circ }34'84.6''E$). We identified the *misinformation* dataset by extracting a subset of our downloaded tweets that contained the terms “misinformation”’, “false”, “fake” or “lie”, directly reported by people in their tweets. These were tweets in which a user either identified information or other messages as misinformation or questioned whether that message or information was misinformation. We also identified tweets related to *healthcare* information by extracting those tweets containing the key terms “hospital” or “test”. To preprocess these data we removed punctuation marks and uniform resource locators (urls) using the tidytext package^50^, and we replaced abbreviations, symbols, contractions, ordinals and numbers with the words they represent using the qdap package^51^. tidytext was also used to unnest the unigrams (single words) and bigrams (sequential word pairs) from each tweet. Lastly, word stemming was conducted using the wordStem function of the SnowballC package (https://cran.r-project.org/web/packages/SnowballC/SnowballC.pdf) for being able to score affine words in terms of positivity rather than disregarding these words.

### Epidemiological data mining and preprocessing

At the time of our study, epidemiological data was not available for New Delhi specifically (i.e. the case study shown in this paper) nor for local hospitals within the analyzed domain, but rather for the state of Delhi, i.e. the National Capital Region (NCR). The dataset^52^ contained both crowd-sourced and official data from the Ministry of Health and Family Welfare. It included the number of cases and cured, discharged or migrated individuals in the state since March 15, 2020 when India registered its first case. For these motivations we calculated the new daily cases $\Delta I = I(t) - I(t-1)$ where *I* stands for cases, and new hospitalization as $\Delta H = H(t) - H(t-1)$ where hospitalization $H(t) = I(t) - R(t)$ are cases minus the number of patients cured, discharged or migrated. Later we located hospital level data from information reported by the New Delhi from the Ministry of Health and Family Welfare (https://coronabeds.jantasamvad.org) which indicated the daily number of hospital beds occupied within a geo-located area. The vast majority of these hospitals resulted to be private hospitals. We conducted validation of our spatio-temporal forecasting model by comparing city-scale calculated hospitalization versus hospital-scale data for the same city. As for Mumbai, the situation was analogous to New Delhi; data of cases and hospitalization was only available at the state scale, i.e. Maharashtra. Thus, cases and hospitalization of Mumbai was calculated as $\sim 50\%$ of the whole state as evidence supported.

### Sentiment quantification

Sentiment analyses performed for InTo involved quantifying both categorical emotions and positivity of each text corpus given unigrams (words) within extracted tweets. The labtMT lexicon^1^, accessed via the qdap package, was used to measure the positivity and the nrc lexicon^53^, accessed via the tidytext package, was used to evaluate emotional affects (or categories) in a tweet. The continuous (real number) positivity of a tweet (*P*) was quantified as:

5.1 $$\begin{aligned} P= \sum _{i=1}^{N}p_{avg}(w_{i})\cdot \frac{f_{i}}{\sum ^{N}_{j=1}f_{i}} \end{aligned}$$

where $p_{avg}(w_{i})$ is the positivity value of each word $(w_{i})$ as indicated in the labMT lexicon, and $f_i$ is the frequency of each word. The daily positivity ($\bar{P_t}$), given $N_t$ number of tweets on day *t* is calculated by

5.2 $$\begin{aligned} \bar{P_t} = \frac{\sum _{j=1}^{N_{t}}P_{j}}{N_{t}} \end{aligned}$$

where *j* is indicating all tweets in the day considered. The emotion of a tweet was considered to be the distribution of the affect categories (for example, anger, surprise, joy, etc.) associated with each word of a tweet. We noted the affect categories associated with each unigram and then counted the number of times each affect category appeared in a tweet and in a day. Weekly calculations of positivity and emotion categories are calculated considering average value of sentiments at the weekly scale.

### Forecasting

#### ARIMA temporal forecasting

InTo perform weekly temporal forecasts of new cases and hospitalizations as a function of tweet positivity and historical epidemiological events. A two-step non-seasonal ARIMA(*p*, *d*, *q*) model is used for temporal forecasting where parameters *p*, *d*, and *q* are non-negative integers; *p* is the order (number of time lags) of the autoregressive model considering long term trends (e.g. seasonality), *d* is the degree of differencing (the number of times data are subtracted to past values) that considers memory for non-seasonal events, and *q* is the order of the moving-average model for errors establishing their temporal impact. The ARIMA model was selected due to the validated linear patterns between positivity and hospitalization as well as positivity and cases at the weekly scale. Because (*p*, *d*, *q*) parameters and coefficients are updated weekly in order to optimize forecasts, the model can be considered dynamically ”non-linear” in the parameter space despite its linear formulation. Temporal forecasts were calculated using a non-seasonal ARIMA model as implemented in the fable package^54^. The two-step forecast is done because first positivity is forecasted for the week following the one considered and after cases and hospitalization are forecasted based on future positivity. The analytic form of the ARIMA model is written for $y = \Delta H$ as new hospitalization that is the primary target of InTo; however, *y* can generally be positivity or cases based on the selected predictand. Thus, hospitalization is forecasted as:

5.3 $$\begin{aligned} \Delta H^d_t = \beta _0 + \beta _1{\bar{P}}_t + \phi _1\Delta H^d_{t-1} + \cdots + \phi _p \Delta H^d_{t=p} + \theta _1\varepsilon _{t-1} + \cdots + \theta _q\varepsilon _{t-q} + \varepsilon _t \end{aligned}$$

where $\Delta H$ is differenced to an order of *d* (not that *d* is an index and not a power exponent), $\beta _0$ is a constant, $\beta _1$ is the regression coefficient for average positivity ${\bar{P}}_t$, $\phi _1y^d_{t-1} + \cdots + \phi _py^d_{t=p}$ is an autoregessive model of order *p* and $\theta _1\varepsilon _{t-1} + \cdots + \theta _q\varepsilon _{t-q} + \varepsilon _t$ is a moving average model of order *q*. The error terms $\varepsilon _t$ of $\Delta H$ are assumed to be independent and identically distributed sampled from a normal distribution with zero mean. Thus, $\varepsilon _t$ is a white noise factor.

Default settings of the ARIMA function in the fable package was selected as it automatically determines the values of *p*, *d* and *q* that minimize the Akaike Information Criterion (AIC). We retrained our model weekly, using the entire history of positivity and epidemiological data to date. We utilize an ex-post forecasting approach where we first project the next week’s values of positivity by applying the ARIMA model to tweet positivity. The ARIMA model is of a similar form to Eq. 5.3, except that positivity is the outcome value and the $\beta _1{\bar{P}}_t$ term is excluded. Following this we used the ARIMA model to forecast cases and hospitalizations considering the ARIMA linearized relationship between the history of epidemiological factors and tweet positivity and the projected values of positivity. Equivalently, without altering the ARIMA structural form in Eq. 5.3, we predicted new hospitalization considering different predictands, i.e. tweet volume, volume and positivity, or hospitalization only to select the optimal model with the highest prediction accuracy.

We conducted a validation exercise to evaluate the performance of this modeling approach (see Fig. 8). We split the data into a training and test set such that the training set included a week of data and the test set contained the same data as the training set and an additional week of data that did not appear in the training set. Four models were then trained on the training set using different predictors: using the epidemiological data alone to forecast itself (ACF); using positivity to forecast epidemiological data; using tweet volume to forecast epidemiological data; and using positivity and tweet volume to forecast epidemiological data. The models were then used on the test set and the results were compared to the observed data. This process was repeated with the training set and test sets being increased by one week until the entire data set was used. The mean absolute percentage error (MAPE) of each model was computed to quantify the accuracy of the model: the lower the MAPE, the more accurate the model.

#### Geostatistical forecasting

We predicted the spatial spread of healthcare pressure with geostatistical kriging considering the inferred linear relationship between positivity and cumulative hospitalization at the city scale. This relationship is linked to the $\beta _1$ exponent in the ARIMA model of Eq. 5.3 and is updated every week. A similar modeling was performed in the past by Berke, O.^55^. Geostatistical kriging was performed using the automap package^56^, and the results were visualised as a heatmap overlaying satellite imagery obtained from Google Maps using the ggmap package version 3.0.0^57^ . We restricted data to the most recent two weeks of tweets and cumulative hospitalization to ensure that there was enough geo-spatial tweet data salient to predict the last observed hospitalization. This was also supported by the limited “memory” of positivity for hospitalization, reflected by low values of the ARIMA parameters *p* and *d*. As with the double step prediction of ARIMA, first we extrapolate positivity over the whole geographical domain and after we perform a second geokriging to predict new hospitalization based on the positivity-hospitalization relationship. Given the limited volume of geo-located tweets, we used ordinary geo-statistical kriging because average is likely constant^58^ (as in our case) to interpolate positivity using the semi-variogram:

5.4 $$\begin{aligned} \gamma _{P}(\delta )= & {} \frac{1}{2N(\delta )}\bigg \{\sum ^{N(\delta )}_{i=1} {[}P_{i+\delta }-P_i]^2\bigg \} \end{aligned}$$

5.5 $$\begin{aligned} \hat{P_j}= & {} \sum _{i=1}^{M}\lambda _i(P_j) \cdot P_i \end{aligned}$$

where $\lambda _i(P_j)$ is a kriging weighting factor for the know value of the variable *P* at a sampled location *i* and $j \ne i$. A function is a semivariogram only if it is a conditionally negative definite function, i.e. for all weights $\lambda _1, ..., \lambda _M$ subject to $\sum _{i=1}^M \; \lambda _i(P_j)=0$ and locations *i*, ..., *M* it holds: $\sum _{i,j=1}^M \; \lambda _i \; \gamma _{P}(i,j) \; \lambda _j$. This establishes the connection between predictions of Eq. 5.5. and semivariogram of Eq. 5.4. The experimental semi-variogram of the data at the observation location is fitted against a theoretical semi-variogram model of ${\hat{\gamma }}_{P}(\delta _P)$; the latter is an exponential, Gaussian or spherical semivariogram. One is thus making a distinction between the experimental variogram that is a visualization of the observed possible spatio-temporal correlation and the variogram model that is further used to define the weights of the kriging function on which predictions are based. *M* is the number of (10,000) randomly generated points which are interpolated using the kriging weighting factor $\lambda _i(P_j)$ determined by the semivariogram.

Next, we applied universal geostatistical kriging^59^ to interpolate the expected hospitalization ${\hat{H}}$ over space considering the forecast based on the relationship between twitter positivity and the state-level cumulative hospitalization. Universal kriging is used because it assumes that the average is not constant as it is in our case. This is done by using the following analytics:

5.6 $$\begin{aligned} {\hat{\gamma }}_{H}(\delta )= & {} \frac{1}{2N(\delta )}\sum ^{N(\delta )}_{i=1}[({\hat{H}}_{i + \delta } - m) - ({\hat{H}}_i - m)]^2 \end{aligned}$$

5.7 $$\begin{aligned} \hat{H_j}= & {} m + \sum ^M_{i=1}\lambda _i(H_j) \cdot ({\hat{H}}_i - m) \end{aligned}$$

where ${\hat{\gamma }}_{H}(\delta )$ is the predicted semivariogram of expected positivity based on $m({\hat{P}}) = \sum ^L_{l=0}\alpha _l \; f_l({\hat{P}})$ that is a slow and continuous trend function^60^ capturing the linear relationship between hospitalization and tweet positivity among points *l*; these points may be different from the whole set of points *M* over which interpolation is performed. Finally, to determine the healthcare pressure $H_P$ at each point *i* we used

5.8 $$\begin{aligned} H_{P_i} = {\left\{ \begin{array}{ll} {\hat{H}}_i - \langle \hat{H_T} \rangle = {\hat{H}}_i - \frac{\sum _{i=1}^M \hat{H_i}}{M} &{} \text {if} > 0\\ 0 &{} \text { otherwise} \end{array}\right. } \end{aligned}$$

where $\langle \hat{H_T} \rangle$ is the expected average of hospitalization over the selected geographical domain, and *M* is the number of interpolated points. We applied the same model to spatially explicit hospital bed occupancy in order to compare interpolations of hospitalization based on state and hospital level data.

### Predictability indicators

Weekly indices are introduced to monitor the evolution of the pandemic, the short- and long-term predictability of Twitter positivity and the departure between forecasts and observations. The *Risk Index* is set to measure the rate of change in epidemiological values, yet in formulating indication of epidemic trends. The *Gap Index* is introduced as the difference between forecast predictions and observations normalized to previous observations. The *Correlation Index* is calculated by estimating the Pearson correlation coefficient to quantify the short-term forecast ability of positivity for epidemiological variables (new hospitalizations and new cases) via geokriging over space and via ARIMA over time. The first ARIMA component and geokriging factors are linear functions of the linearized relationship between positivity and epidemiological variables (Eqs. 5.3 and 5.7). To quantify the long-term predictability of highly diverging events, transfer entropy (TE) as in Li and Convertino^28^ is introduced as the *Predictability Index* that informs about the probabilistic predictability of positivity for epidemiological patterns in terms of probability distribution functions (pdfs) rather than time point values. The higher TE, the higher the time-delayed and/or divergent influence (as pdfs) of positivity for hospitalization or cases. We did not use TE for predictive purposes because we never forecasted events beyond one week within which the socio-epidemiological linearity holds. All indices are analytically defined as:

5.9 $$\begin{aligned} R(Y_t)= & {} (y_{t} - y_{t-1})/y_{t-1}= \Delta Y(\delta t)/100 \end{aligned}$$

5.10 $$\begin{aligned} G(Y_t)= & {} \hat{y(t)} - y(t) / y(t) = \Delta {\hat{Y}}_t/100 \end{aligned}$$

5.11 $$\begin{aligned} \text{ corr }(P,Y)= & {} \frac{\sum _{t=1}^L(p_t-{\bar{P}})(y_t-{\bar{Y}})}{\sqrt{\sum _{t=1}^L(p_t-{\bar{P}})^2\sum _{t=1}^L(y_t-{\bar{Y}})^2}} \end{aligned}$$

5.12 $$\begin{aligned} TE_{P \rightarrow Y}= & {} \sum {p}(Y_{t}, Y_{t-1}, P_{t-1})\cdot \text{ log } \Bigg (\frac{p(Y_{t}|Y_{t-1}, P_{t-1})}{p(Y_{t}|Y_{t-1})}\Bigg ) \end{aligned}$$

where $Y=I \;\; \text{ or } \;\; \Delta H$ is indicating time series of cases or hospitalization, respectively, and *y* indicates time point values. ${\bar{P}} = \frac{1}{L} \sum _{t=1}^L p_t$ and ${\bar{Y}} = \frac{1}{L} \sum _{t=1}^L y_t$. *L* is the length of time-series of P and Y.

The Value of Misinformation (*VoMi*) was defined as the difference of gap indices as:

5.13 $$\begin{aligned} VoMi(t) = G(Y_t)_{S} - G(Y_t)_{M} \end{aligned}$$

where *S* and *M* stand for the systemic Twitter information and classified misinformation set in predicting *Y* as cases or hospitalization. VoMi provides users with a measure of how misinformation impact forecasts of epidemiological variables with respect to the systemic tweet information considering both model and data uncertainty contained in the gap index.

Increasing values of VoMi (independently of the sign) indicate that the misinformation tweet subset has increasing importance in forecasting versus the full tweet set. On average, if VoMi is positive, misinformation does contribute non-negligibly to overpredict epidemiological trends, whereas if it is negative it impacts positively and substantially the forecasts proportionally to the magnitude of the misinformation gap $G(Y_t)_{M}$. This is evaluated for the same model structure and epidemiological data uncertainty of the full tweet information. It should be noted that both gap indices $G(Y_t)_{S}$ and $G(Y_t)_{M}$ can be negative and $M \subseteq S$, yet the relative (non-linear) balance between full information and misinformation (positivity) predictability contribute to determining VoMi.

In a decision analytical sense VoMi is defined as the amount of resources a decision maker would be willing to pay for extra information that increase forecast accuracy before an event occurs. The optimal information set $\mathbf{I} _{opt}$ is defined as the one whose gap is minimized (assuming that data are perfect “error-free” information to match) and equal to $G(\mathbf{I} _{opt}) = G(\mathbf{I} _{sub}) - VoMi(\mathbf{I} _{opt},\mathbf{I} _{sub})$ where $VoMi = MI(P,H)$ that is the mutual information $MI(P,Y) = \sum _p \sum _y \; p(p,y) \; \text{ log }\frac{p(p,y)}{p(p)p(y)}$ between positivity and cases or hospitalization. Mutual Information in an information-theoretic variable measuring the amount of information shared between two variables that is on average inversely proportional to the predictability indicator in Eq. 5.12 (i.e. the uncertainty reduction between variables).

### Tweet spread

For each week, we calculated and displayed the average daily tweet and retweet volume for all tweets and the misinformation related tweets. Time series of tweet and retweet volumes, as well as their corresponding average positivity, are displayed by InTo, which serve as indicators of spreading potential of COVID-19 related messages within and beyond the geographical domain considered. Additionally, the Twitter user of the most popular tweet in a week is shown when hovering over a point on the Tweet spread plot.

### Dashboard architecture

The InTo dashboard utilizes a client-server architecture designed and implemented using the shiny package^61^ in R^62^ that provides a convenient wrapper for interactive HTML widgets. This is similar to GLEaMviz architecture^63^. The client component only allows users to visualize the results of InTo but many outputs, for example predictability indicators, are downloadable by users. All computations on the server are conducted in R using the established workflow (see Fig. 1).

## InTo online

InTo online dashboards and data are at:

https://nexuslab.shinyapps.io/InTo_Delhi/ for the city of New Delhi

https://nexuslab.shinyapps.io/InTo_Mumbai/ for the city of Mumbai

Into online manual, workflow, data sources and codes is at:

https://rpubs.com/elroyg1/Into-walkthrough

Into main code is at:

https://github.com/elroyg1/InTo.

## Data ethical approval

Twitter data are collected by leveraging Twitter’s free streaming API. A Twitter developer account was obtained as well as the necessary authentication tokens. The data set is available in compliance with the Twitter’s Terms and Conditions (https://developer.twitter.com/en/developer-terms/agreement-and-policy), under which we are unable to publicly release the text of the collected tweets. Twitter developer account was obtained on May 7, 2020. We are, therefore able to release Tweet IDs, which are unique identifiers tied to specific tweets. The Tweet IDs can be used by researchers to query Twitter’s API and obtain the complete tweet object, including tweet content (text, URLs, hashtags, etc) and authors’ metadata. Our collection relies upon publicly available data (both epidemiological and Twitter data) and is hence registered as IRB (institutional review board) exempt by Hokkaido University.

Satellite images were obtained from Google Maps using their Maps Static API. Our use is in compliance with Google’s Google Maps/Google Earth Additional Terms of Service, which allows us to view and annotate maps, as well as publicly display content with proper attribution online and in print for non-commercial use (https://www.google.com/help/terms_maps/).

## Supplementary information

Supplementary Information.
