# Supplementary material for: In.To. COVID-19 socio-epidemiological co-causality
Source: Sci Rep. 2022 Apr 6;12:5831. doi: 10.1038/s41598-022-09656-1 (PMC8986029; doi:10.1038/s41598-022-09656-1)
Supplement: Supplementary file 1 — Supplementary Information. [file 41598_2022_9656_MOESM1_ESM.pdf]

Supplementary Figure Captions

Figure S1. Spatial and Temporal Forecasts for Mumbai. The results of spatial and temporal forecasts of healthcare pressure for Mumbai between May and August 2020 are displayed. Gradients of healthcare pressure are determined by variability of estimated cases (over the average) across space.

Figure S2. Emotions, Top Bigrams and Tweets for Predictive Information, Misinformation and Healthcare from Mumbai. The time series of emotions and the top word pairs and tweets from the systemic information (all tweets) for Mumbai are shown. On the left are displayed the tweet texts ordered in ascending order by tweet positivity. The top 5 word pairs for August 13 are displayed at the bottom.

Figure S3. Predictability Indicators for Mumbai. Predictability indicators for Mumbai.

Figures S4. Information Volume and Spreading Potential for Mumbai. The spread of all tweets and misinformation-related tweets for Mumbai are shown. On the right, the text of the most popular tweets from Mumbai are displayed.

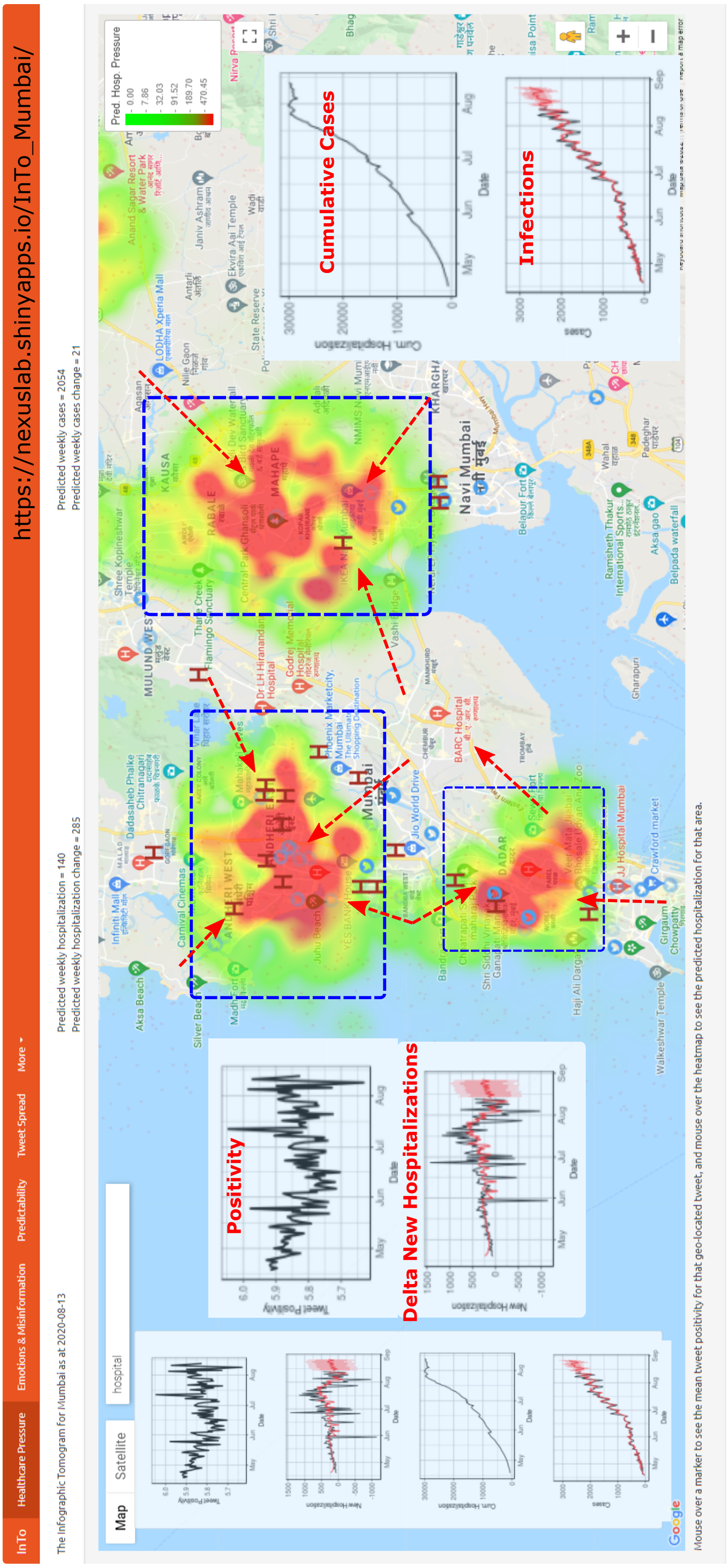
https://nexuslab.shinyapps.io/InTo_Mumbai/
InTo
Healthcare Pressure
Emotions & Misinformation
Predictability
Tweet Spread
More
The Infographic Tomogram for Mumbai as at 2020-08-13
Predicted weekly hospitalization = 140
Predicted weekly hospitalization change = 285
Predicted weekly cases = 2054
Predicted weekly cases change = 21
Map
Satellite
hospital
Pred. Hosp. Pressure
Cumulative Cases
Infections
Positivity
Delta New Hospitalizations
Mouse over a marker to see the mean tweet positivity for that geo-located tweet, and mouse over the heatmap to see the predicted hospitalization for that area.

Figure S1:

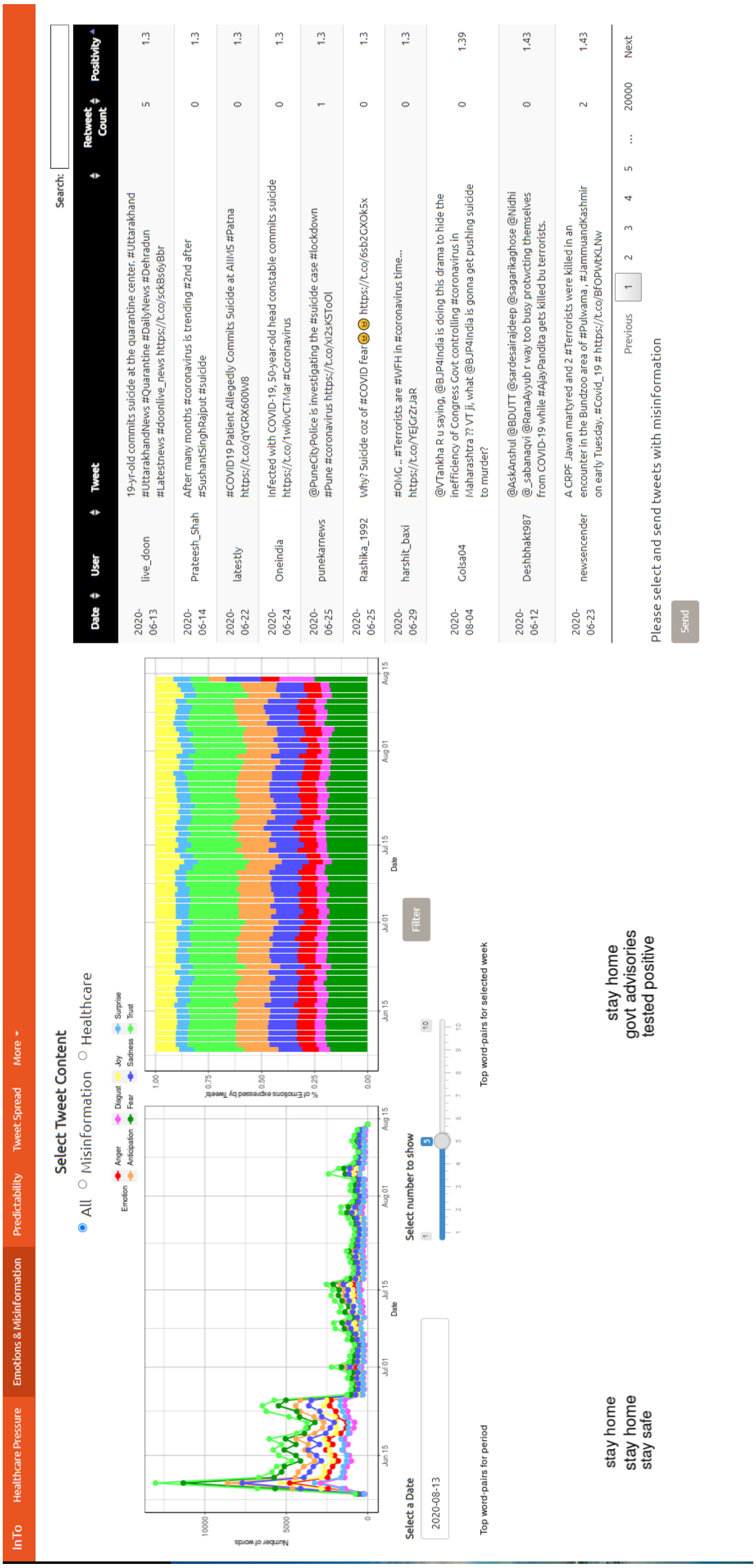
InTo
Healthcare Pressure
Emotions & Misinformation
Predictability
Tweet Spread
More
Select Tweet Content
All
Misinformation
Healthcare
Emotion
Anger
Anticipation
Disgust
Fear
Joy
Sadness
Surprise
Trust
Number of words
% of Emotions expressed by Tweets
Date
Select a Date
2020-08-13
Select number to show
Filter
Top word-pairs for period
Top word-pairs for selected week
stay home
stay home
stay safe
stay home
govt advisories
tested positive
Search:
Date
User
Tweet
Retweet Count
Positivity
2020-06-13
live_doon
19-yr-old commits suicide at the quarantine center. #Uttarakhand #UttarakhandNews #Quarantine #DailyNews #Dehradun #Latestnews #doonlive_news https://t.co/sckBs6yBbr
5
1.3
2020-06-14
Prateesh_Shah
After many months #coronavirus is trending #2nd after #SushantSinghRajput #suicide
0
1.3
2020-06-22
latestly
#COVID19 Patient Allegedly Commits Suicide at AIIMS #Patna https://t.co/qYGRX600W8
0
1.3
2020-06-24
Oneindia
Infected with COVID-19, 50-year-old head constable commits suicide https://t.co/1wl0vCTMar #Coronavirus
0
1.3
2020-06-25
punekarnews
@PuneCityPolice is investigating the #suicide case #lockdown #Pune #coronavirus https://t.co/Xl2sK5TOOl
1
1.3
2020-06-25
Rashika_1992
Why? Suicide coz of #COVID fear https://t.co/6sb2CXOk5x
0
1.3
2020-06-29
harshit_baxi
#OMG.. #Terrorists are #WFH in #coronavirus time... https://t.co/YEjGrZrJaR
0
1.3
2020-08-04
Colsa04
@VTankha R u saying, @BJP4India is doing this drama to hide the inefficiency of Congress Govt controlling #coronavirus in Maharashtra ?? VT ji, what @BJP4India is gonna get pushing suicide to murder?
0
1.39
2020-06-12
Deshbhakt987
@AskAnshul @BDUTT @sardesairajdeep @sagarikaghose @Nidhi @_sabanaqvi @RanaAyyub r way too busy protecting themselves from COVID-19 while #AjayPandita gets killed bu terrorists.
0
1.43
2020-06-23
newsencender
A CRPF Jawan martyred and 2 #Terrorists were killed in an encounter in the Bundzoo area of #Pulwama , #JammuandKashmir on early Tuesday. #Covid_19 # https://t.co/BfOPWtKLNw
2
1.43
Previous
1
2
3
4
5
...
20000
Next
Please select and send tweets with misinformation
Send

Figure S2:

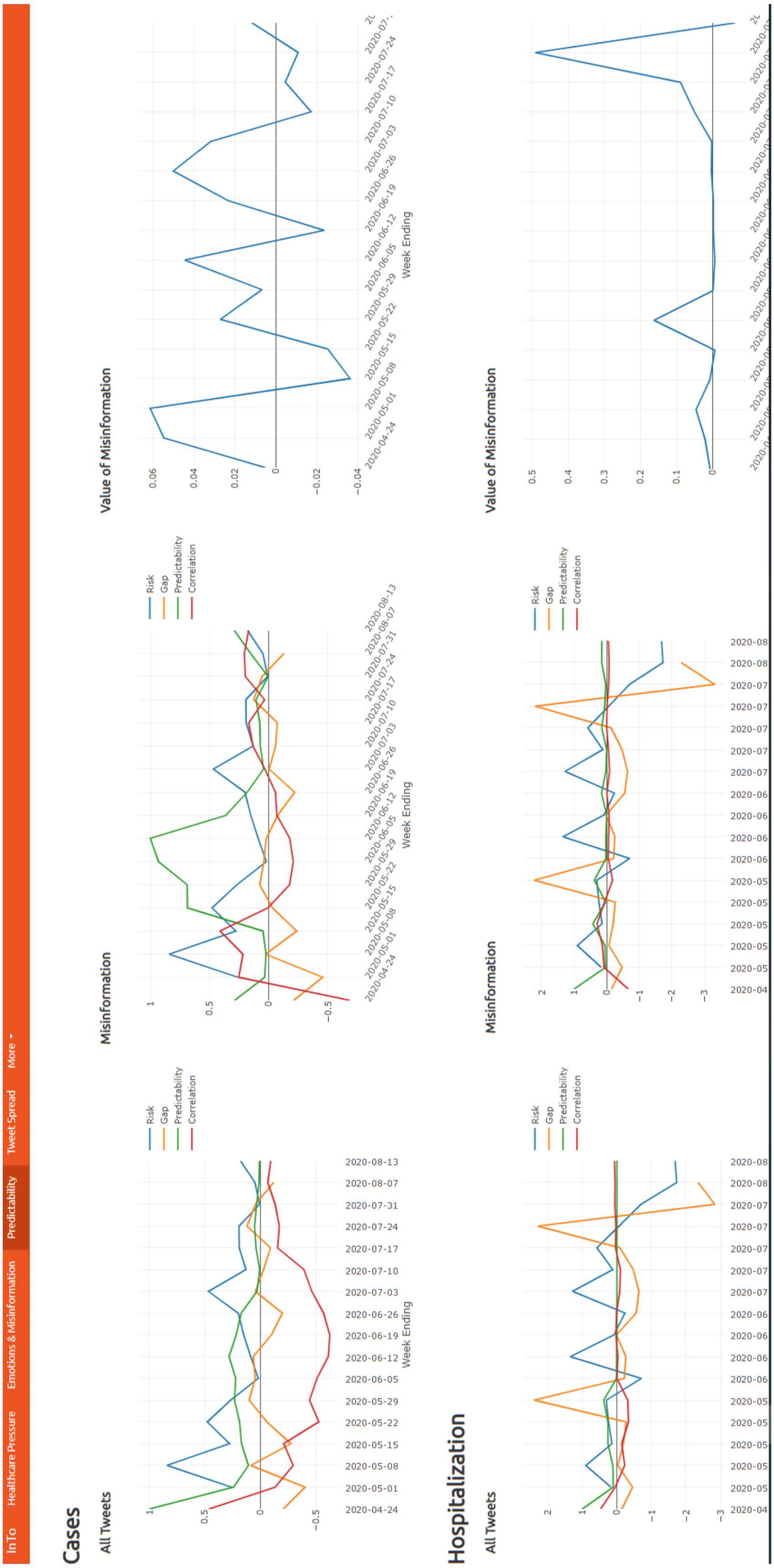
InTo
Healthcare Pressure
Emotions & Misinformation
Predictability
Tweet Spread
More
Cases
All Tweets
Misinformation
Value of Misinformation
Risk
Gap
Predictability
Correlation
Week Ending
Hospitalization
All Tweets
Misinformation
Value of Misinformation
Risk
Gap
Predictability
Correlation

Figure S3:

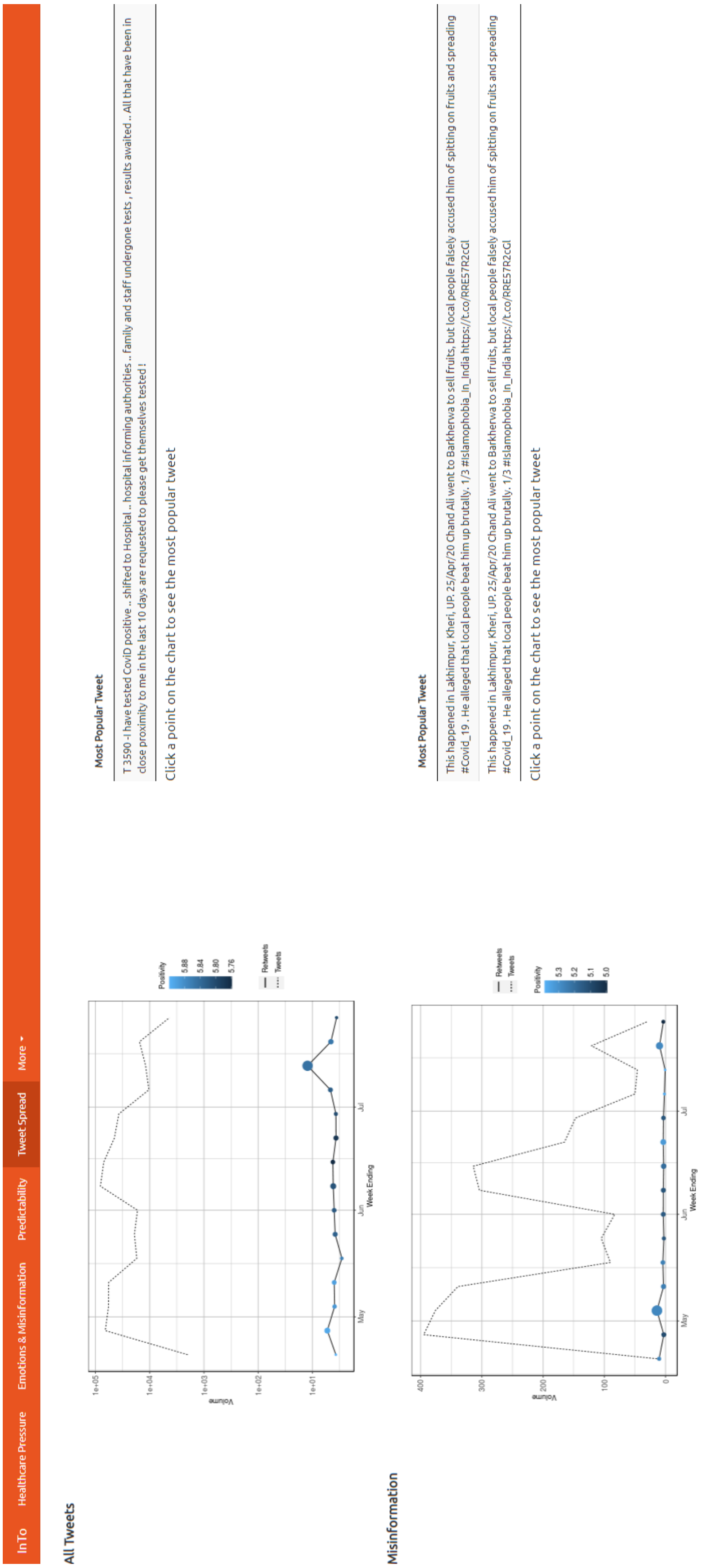
InTo
Healthcare Pressure
Emotions & Misinformation
Predictability
Tweet Spread
More
All Tweets
1e+05
1e+04
1e+03
1e+02
1e+01
Volume
May
Jun
Jul
Week Ending
Positivity
5.88
5.84
5.80
5.76
Retweets
Tweets
Most Popular Tweet
T 3590 -I have tested CoviD positive .. shifted to Hospital .. hospital informing authorities .. family and staff undergone tests , results awaited .. All that have been in close proximity to me in the last 10 days are requested to please get themselves tested !
Click a point on the chart to see the most popular tweet
Misinformation
400
300
200
100
0
Volume
May
Jun
Jul
Week Ending
Retweets
Tweets
Positivity
5.3
5.2
5.1
5.0
Most Popular Tweet
This happened in Lakhimpur, Kheri, UP. 25/Apr/20 Chand Ali went to Barkherwa to sell fruits, but local people falsely accused him of spitting on fruits and spreading #Covid_19 . He alleged that local people beat him up brutally. 1/3 #Islamophobia_In_India https://t.co/RRE57R2cGl
This happened in Lakhimpur, Kheri, UP. 25/Apr/20 Chand Ali went to Barkherwa to sell fruits, but local people falsely accused him of spitting on fruits and spreading #Covid_19 . He alleged that local people beat him up brutally. 1/3 #Islamophobia_In_India https://t.co/RRE57R2cGl
Click a point on the chart to see the most popular tweet

Figure S4:
